# Subcellular Euclidean distance measurements with multicolor fluorescence localization imaging in cultured cells

**DOI:** 10.1016/j.xpro.2021.100774

**Published:** 2021-11-15

**Authors:** Tsvetelina E. Germanova, Emanuele Roscioli, Jonathan U. Harrison, Andrew D. McAinsh, Nigel J. Burroughs

**Affiliations:** 1 Centre for Mechanochemical Cell Biology, University of Warwick, Coventry CV47AL, UK; 2 Division of Biomedical Sciences, Warwick Medical School, University of Warwick, Coventry CV4 7AL, UK; 3 Mathematics Institute, University of Warwick, Coventry CV4 7AL, UK

**Keywords:** Cell Biology, Cell culture, Microscopy

## Abstract

This protocol measures the 3D Euclidean distance (Δ_3D_) between two/three fluorescently labeled kinetochore components in fixed samples using Kinetochore Delta software (KiDv1.0.1, MATLAB based). Overestimation of mean Δ_3D_ is corrected through a Bayesian algorithm, with Δ_EC_ distances reflecting the ensemble average positions of fluorophores within a kinetochore population. This package also enables kinetochore categorization, which can be used to sub-sample kinetochores and measure Δ_EC_. Together, this allows the dynamic architecture of human kinetochores to be investigated (tested in hTERT-RPE1 cells).

For complete details on the use and execution of this protocol, please refer to Roscioli et al. (2020).

## Highlights

• Measurements of intra-kinetochore distances from multi-color image stacks
• Automated detection of fluorescent spot centers with sub-pixel accuracy
• GUI-based pipeline to enable spot classification and data quality control
• Bayesian algorithm to correct for over-estimation of Euclidian distances

## Graphical abstract

**Figure:**
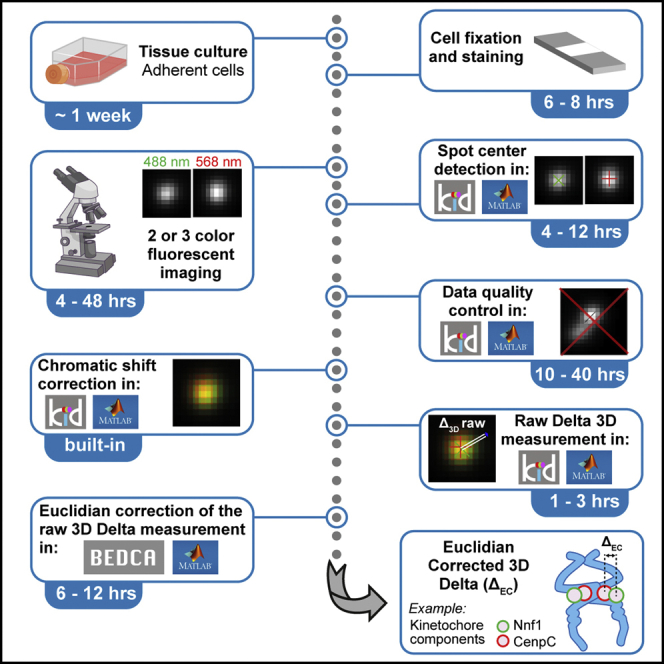

## Before you begin

### Imaging setup requirements

**Timing: 2 h to 1 week**

1. Objective choice: This protocol is developed for measurement of distances between light diffraction limited spots via light microscopy. As such, we aim for imaging at the limit of light microscopy. The resolution as given by the Abbey limit (also known as Rayleigh criterion) and depends on the numerical aperture of the lens. To resolve spots therefore, we used 100x objective with numerical aperture (NA) of 1.4. Objectives with higher numerical aperture can also be used.

***Note:*** The objective is also recommended to be apochromatic such that it largely corrects for the chromatic shift in the sample. However, we use chromatic shift corrections in the software and, therefore, chromatic shift correction by the objective is not critical.

2. Effective pixel size: The sampling in xy-direction is set by the effective pixel size of the microscope. The effective pixel size of the microscope depends on the combination of camera, objective and tube lens. If unknown, the effective pixel size can be calculated by dividing the camera pixel size by the total objective magnification (see https://openwetware.org/wiki/Methods_to_determine_the_size_of_an_object_in_microns). To determine the optimal effective pixel size, i.e., the optimal sampling in the xy direction, use the Nyquist criterion, i.e., the sampling required to get the best resolution possible of the imaging system. The Nyquist sampling can be calculated using the app in the following link: https://svi.nl/NyquistCalculator. In the protocol development we used a spinning disk confocal microscope equipped with 100x objective with 1.4 NA and immersion oil with 1.515 refractive index. In this system we did not use signal amplification and therefore the number of excitation photons is set to 1. **For example:** In the development of this protocol, we utilized a confocal microscope with backprojected pinhole radius of 250 nm. Therefore, in our case the pinhole radius is similar to one Airy disk and we can utilize sampling of 1.6 times higher than the Nyquist limit which results in the following sampling limits: 68.8 nm in xy and 216 nm in z.

***Note:*** In the case of a confocal microscope with the above set parameters, the Nyquist sampling is 43 nm in xy and 130 nm in z. However, the sampling can be increased without significantly compromising the resolution as follows: it can be increased 1.6 times if the confocal microscope has back-projected pinhole size similar to one Airy disk radius; 1.3 times if the back-projected pinhole radius is similar to half of the Airy disk radius; and 2 times if the back-projected pinhole radius is similar to four or more times the Airy disk radius. For description of what an Airy disk is see: https://svi.nl/AiryDisk . For a description of the back-projected pinhole radius of a confocal microscope see: https://svi.nl/PinholeRadius. For online calculator of the back-projected pinhole radius for a confocal microscope of choice see: https://svi.nl/BackprojectedPinholeCalculator. The Airy disk radius is given by the following equation: r$=0.61\frac{\lambda }{NA}$ , where r is the Airy disk radius, λ is the wavelength and NA is the objective numerical aperture. If we calculate the Airy disk for 525 nm wavelength emission, i.e. green channel, we obtain Airy disk radius of 228 nm. The user should calculate the Airy disk radius of their system of choice and then check if the sampling can be increased without compromising the resolution as described above.

***Note:*** If the effective pixel size is not close to optimal, i.e. the effective pixel size is not within about 30% of the optimal pixel size, the user is suggested to choose another imaging system or consult with the imaging specialist responsible for the system to understand what modifications can be made to improve the effective pixel size.

3. Accurate z sampling: motorized piezo stage with nanometer accuracy. The user is highly recommended to utilize motorized piezo stage with nanometer accuracy in the lateral direction. If such stage is not used, the sampling in the lateral (z) direction may be inaccurate and result in improper sample acquisition. For the development of this protocol, we utilized PSZ400-S which has resolution of ~6 nm.
4. Sample drift: The user is recommended to check for noticeable sample drift in the imaging system of choice. This can be done by using fluorescently labeled beads, or one of the sample preparations described later in the protocol. Here, the user is to position the sample and take images every second over several seconds (or similar). Upon maximum projection of the acquired images, the spots should co-localize. If visually, the spots in the projection appear smudged in a single direction, the user should consult with imaging specialist for sample drift on the microscope.
5. The user is recommended to use deconvolution on the acquired images. Deconvolution of images relates tightly to the imaging system of choice and therefore the user is recommended to consult with the imaging specialist maintaining the microscope or the company.

### Software installation in MATLAB

**Timing: 2–6 h**

***Note:*** the software has been developed on Mac OS (latest version checked is Catalina) and not all user interfaces display fully on Windows, such as the Graphical User Interface (GUI) for jobset setup, steps 3 and 4. The user is recommended to use Mac OS, if possible. If that is not possible, the user can utilize the images in the protocol to navigate in the GUIs. Computer RAM memory is recommended to be 16GB or more. 500GB or more storage space (local, server, or Drive) is recommended.

6. Install MATLAB: First download MATLAB. MATLABR2020a is the latest version on which the software has been tested, and thus is recommended. During MATLAB installation the user will be prompted to select the packages to be installed. In the installation, check the boxes for the following packages: MATLAB; Control System Toolbox; Curve Fitting Toolbox; Deep Learning Toolbox; DSP System Toolbox; Global Optimization Toolbox; Image Processing Toolbox; Instrument Control Toolbox; Optimization Toolbox; Parallel Computing Toolbox; Partial Differential Equation Toolbox; Signal Processing Toolbox; Simulink; Statistics and Machine Learning Toolbox; Symbolic Math Toolbox

***Note:*** If you already have MATLAB version R2020a or later installed proceed to step b.

***Note:*** In later versions of MATLAB the toolboxes may be re-named. If you cannot find one of the toolboxes check in MathWorks for alternative name.

***Note:*** Once MATLAB is installed, a “MATLAB” named folder will appear in the Documents folder on your computer.

7. Check what packages are installed. In the command window type ver and press **Enter.** If not all of the packages stated in a. are installed, navigate to the MATLAB tab Home -> Environment -> Add ons -> Get Add ons. MATLAB will prompt up a window named Add-On Explorer. In the Search field of the window type the name of the missing package and press **Enter.** Navigate to the correct package and press Install. Repeat these steps for all packages that need to be added.

***Note:*** Every time the user types a command (here highlighted in different font) in the Command window, the user should press **Enter** to execute the command.

8. Add KiDv1.0.1 and BEDCA Software to MATLAB.
  a. Download KiDv1.0.1 and the BEDCA software from the following link https://github.com/cmcb-warwick/KiD-also-known-as-KiT2.1.10-and-BEDCA in “.zip” format. Extract the content of the downloaded folder. Open the folder and copy the “KiDv1.0.1” and “BEDCA” folder into the “MATLAB” folder, situated in the “Documents” folder on the computer (path Documents\MATLAB).
  b. Allow MATLAB to access the code: Open MATLAB, and within the Current Folder panel, locate the “KiDv1.0.1” folder, right-click on it and select Add to Path -> Selected Folders and Subfolders. Do the same for the “BEDCA” folder.

**CRITICAL:** Every time the user closes and re-opens MATLAB when doing this protocol, the user should add the “KiDv1.0.1” and “BEDCA” folders to the MATLAB path as described in this step.

### Recommended controls

**Timing: 1–2 weeks**

**Figure 1:**
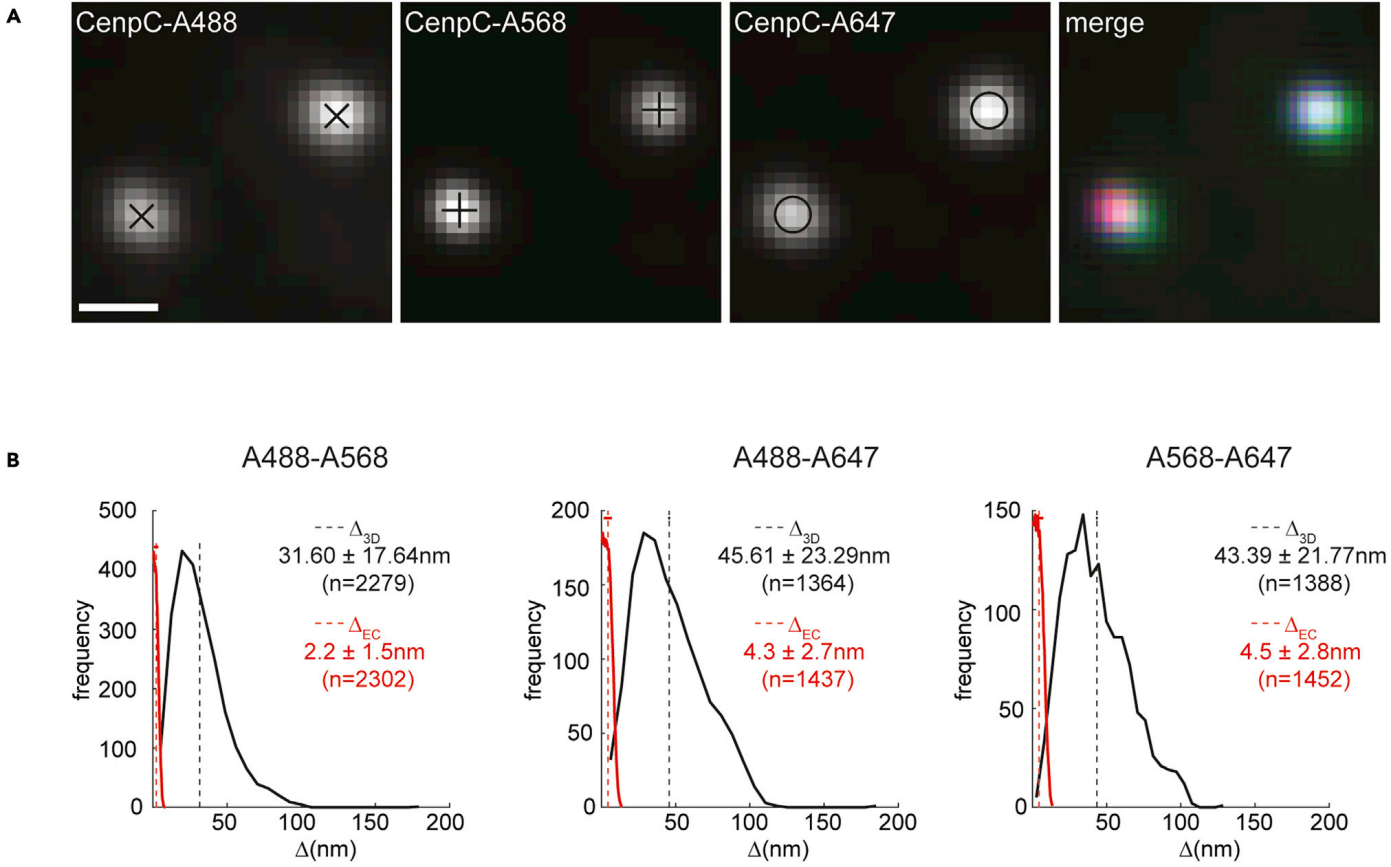
3D Delta Euclidian distance measurements of CenpC to CenpC (0 nm true distance) Comparison between the measurement before and after BEDCA inflation correction. (A) Example images of CenpC labeled with anti-CenpC primary antibody and mix of A488, A568 and A647 secondary antibodies. Scale bar 500 nm. (B) Delta 3D distance measurements of the CenpC-A488-to-CenpCA568, CenpC-A488-to-CenpC-A647, and CenpC-A568-to-CenpC-A647 distances. The measured 3D Euclidian distance distribution (Δ3D) prior to BEDCA distance inflation correction is shown in black. The 3D Euclidian distance distribution after BEDCA distance inflation correction (ΔEC) is shown in red. Mean, standard deviation and sample size for each distribution is indicated on the right. (Adapted from Roscioli et al., 2020 with permission)

***Note:*** The true Delta distance is estimated to be 0 nm for labeling of a single primary antibody with multiple secondary antibodies in different colors because in each color the fluorescent spot comprises multiple secondary antibody molecules that are randomly distributed in the localization zone (the protein labeling can be imagined as a bouquet with randomly distributed colors within). Thus, although at a molecular level two secondary antibodies cannot be exactly colocalized, on average the spot centers of each fluorophore population will be colocalized.

9. Software functionality control: We recommend for the user to download the provided dataset from the following link: https://omero.warwick.ac.uk/webclient/?show=project-4752. The provided datasets have total size of about 7.5 GB. The user can find instructions on how to log-in and download the data on the following page: https://warwick.ac.uk/fac/sci/med/research/biomedical/facilities/camdu/publicdata/. The provided dataset has been published in Roscioli et al., 2020. The “Approximate chromatic shift correction images” data contains hTERT RPE1 cells stained with anti-CenpA primary antibody, that is then labeled with mixture of AlexaFluor 488 (A-488), AlexaFluor 568 (A-568), and AlexaFluor 647 (A-647) secondary antibodies. The “hTERTRPE1 stained for Nnf1/CenpC/Ndc80N” data contains hTERT RPE1 cells stained with anti-Nnf1, anti-CenpC, and anti-Ndc80N primary antibodies that are then labeled with A-488, A-568, and A-647 secondary antibodies, respectively. Then follow the protocol for measuring three-channel Delta measurement, or the desired functionality. That will ensure that the software is running properly on the user computer. The examples in that protocol follow analysis of the provided dataset where possible.
10. Experimental control: We recommend the user to stain the same kinetochore marker in three colors and measure the Delta distance between all pairs of fluorescent channels. Here, the user is recommended to stain a kinetochore marker with one primary antibody and an equal mixture of secondary antibodies labeled in three different colors (A-488, A-564, and A-647). In this experiment, the primary antibody will be recognized by the three types of labeled secondary antibodies, and as the mixture is equal, equal affinities are expected (note that single primary antibody is recognized by many secondary antibodies). Therefore, the center of fluorescence should be at the same place for the three fluorescent secondary antibody populations and the true Delta distance is estimated to be 0 nm (see Note). The expected results for this control are in the range of 0–6 nm distance, as determined experimentally. Performing this control will ensure the immunofluorescence protocol used by the user does not significantly affect the Delta distances measured, as previous study suggested that sample preparation can affect Delta measurements (Suzuki et al., 2018). See Figure 1 for the results of that experiment performed in Roscioli *et al.*

## Key resources table

**Table:** 

REAGENT or RESOURCE	SOURCE	IDENTIFIER
**Antibodies**		

Guinea pig polyclonal anti-CenpC (1:2000)	MBL	Cat#PD030
Mouse monoclonal anti-CenpA (1:300)	Enzo	Cat#ADI-KAM-CC006-E
Mouse monoclonal anti-Hec1 (9G3, 1:1000)	Abcam	Cat#ab3613
Mouse monoclonal anti-Bub1 (1:200)	Abcam	Cat#ab54893
Mouse monoclonal anti-Rod (1:50)	Abcam	Cat#ab56745
Mouse monoclonal anti-Mad1 (F-7) (1:500)	Santa Cruz	Cat#sc-376613
Rabbit polyclonal anti-Nnf1 (1:1000)	McAinsh et al, 2006	n/a
Rabbit polyclonal anti-Knl1 (amino acids 300–350 MELT2, 1:500)	Abcam	Cat#ab70537
Rabbit polyclonal anti-Mad2 (1:500)	BioLegend	Cat#Poly19246
Rabbit polyclonal anti-Knl1-pS24 (1:2200)	Welburn et al., 2010	n/a
Rabbit polyclonal anti-CenpT (1:1000)	Gascoigne et al., 2011	n/a
Rabbit polyclonal anti-Mad1pT716 (1:1000)	Allan et al., 2020	n/a
Goat anti-guinea pig Alexa Fluor 647 (1:500)	Invitrogen	Cat#A21450
Goat anti-guinea pig Alexa Fluor 488 (1:500)	Invitrogen	Cat#A11073
Goat anti-guinea pig Alexa Fluor 568 (1:500)	Invitrogen	Cat#A11075
Goat anti-mouse Alexa Fluor 488 (1:500)	Invitrogen	Cat#A32723
Goat anti-mouse Alexa Fluor 647 (1:500)	Invitrogen	Cat#A21235
Goat anti-mouse Alexa Fluor 594 (1:500)	Invitrogen	Cat# A11032
Goat anti-rabbit Alexa Fluor 488 (1:500)	Invitrogen	Cat#A11008
Goat anti-rabbit Alexa Fluor 594 (1:500)	Invitrogen	Cat#A11037

**Chemicals, peptides, and recombinant proteins**		

VECTASHIELD	Vector	Cat#H-1000

**Deposited data**		

Exemplar data sets	Roscioli et al., 2020	https://omero.warwick.ac.uk/webclient/?show=project-4752.

**Experimental models: Cell lines**		

RPE1 (MC133)	ATCC	Cat#CRL-4000

**Software and algorithms**		

KiDv1.0.1	This paper; Roscioli et al., 2020	https://github.com/cmcb-warwick/KiD-also-known-as-KiT2.1.10-and-BEDCA
BEDCA	This paper; Roscioli et al., 2020	https://github.com/cmcb-warwick/KiD-also-known-as-KiT2.1.10-and-BEDCA
Huygens 4.1 (or other deconvolution software)	SVI	n/a
Matlab (2020a or later)	MathWorks	n/a
Fiji (if required for image stack export to ome.tiff)	Open source	n/a

## Step-by-step method details

In this protocol the user will find instructions how to: (1) Measure intra-kinetochore Delta Euclidian distances between two fluorescently-labeled kinetochore markers – steps 1–6, 9, 10, 12–14; (2) Measure intra-kinetochore Delta Euclidian distances between three fluorescently-labeled kinetochore markers – steps 1–7, 9, 10, 12–14; (3) Measure intra-kinetochore Delta Euclidian distance between two fluorescently-labeled kinetochore markers at kinetochores positive and negative for a third fluorescently-labeled kinetochore marker – steps 1–6, 8, 9, 10, 12–15; and (4) Quantify kinetochore marker intensity – complete step 11 after any of the above analysis.

**Figure 2:**
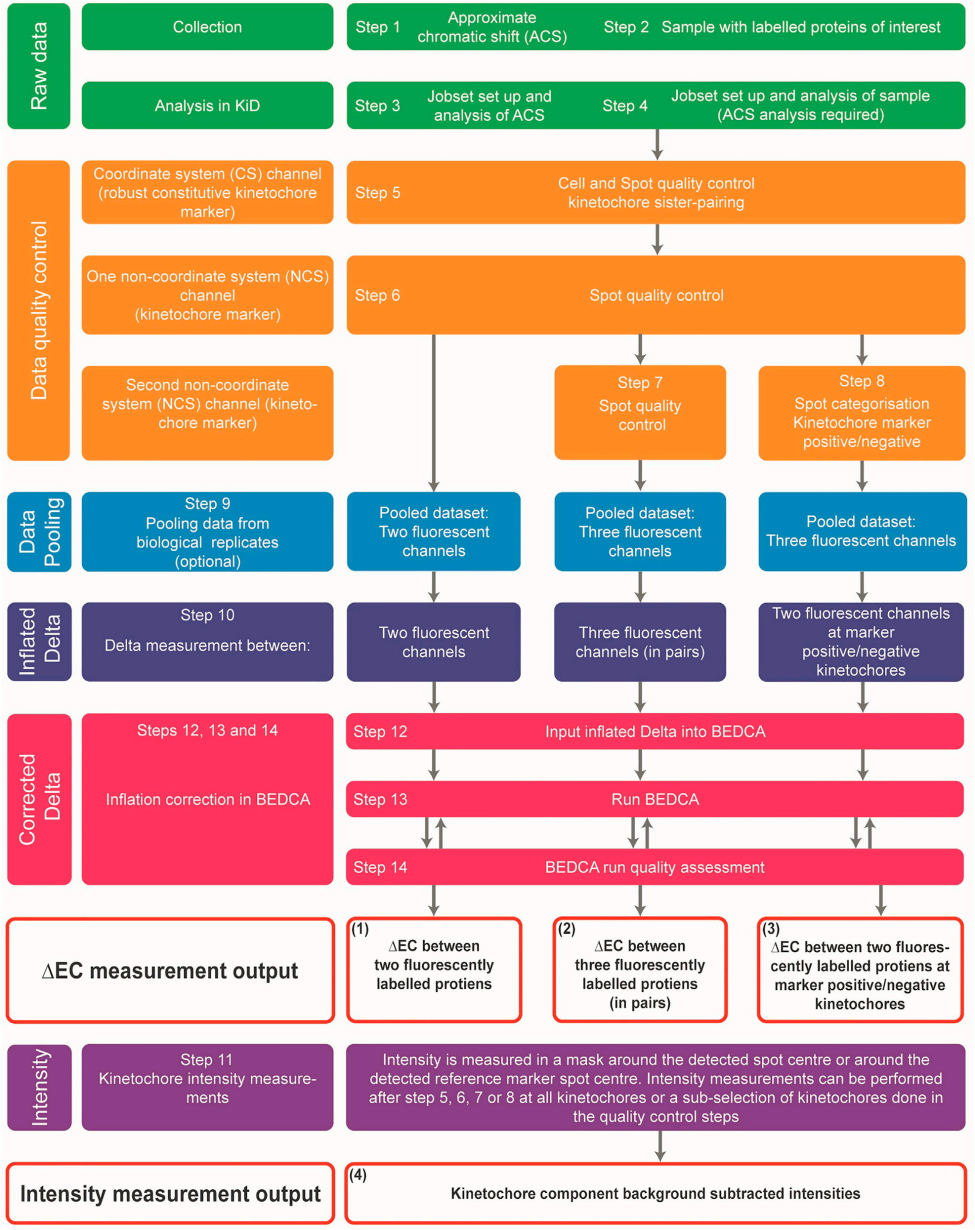
Workflows for Delta Euclidian distance inflation corrected measurements (ΔEC) between kinetochore components and kinetochore intensity measurements The protocol contains the steps for four workflows depending on the desired output: (1) For ΔEC measurement between two fluorescently labeled kinetochore proteins, follow steps 1–6, 9, 10, 12–14. (2) For ΔEC measurements between three fluorescently labeled kinetochore proteins (in pairs), follow steps 1–7, 9, 10, 12–14. (3) For ΔEC measurements between two fluorescently labeled kinetochore proteins at kinetochore marker positive and negative kinetochores, follow steps 1–7, 9, 10, 12–14. (4) For kinetochore intensity measurements, perform step 11 after any of the previous workflows.

For more information on the workflows see Figure 2.

### Raw data collection

**Timing: 1–2 weeks**

This section describes the procedure for collecting the data on the approximate chromatic shift (ACS) correction and the sample of interest: how to grow human adherent cells, prepare immunofluorescent sample, image the sample and export the data.

1. Approximate chromatic shift (ACS) slide preparation and imaging.
  ***Note:*** The ACS analysis (step 3) detects the shift between the kinetochore spots detected in different fluorescent channels for the same marker, i.e. if there is no chromatic shift the spots should perfectly colocalize. Next, in the sample analysis (step 4), the detected sample spot coordinates are corrected for the calculated shift. However, the ACS alone is not sufficiently precise for Delta analysis and further chromatic shift correction is applied in step 10.
  a. Adherent human cell culture: This step provides guidance on how to sub-culture human HTERT-immortalized retinal pigment epithelial (hTERT RPE1) cells. However, other adhesive cell types as well as other organisms can also be potentially used.
    ***Note:*** this is a standard protocol for culturing human hTERT RPE1 cells used for the development of this protocol. However, the user can also use media variations and acquire the reagents from other companies.
    i. Maintenance of hTERT RPE1 cells: cells should be grown in a humidified incubator at 37° and 5% CO_2_. The cells are seeded and grown in T25 or T75 single use sterile flasks containing Dulbecco′s Modified Eagle′s Medium/Nutrient Mixture F-12 Ham (DMEM/F12), supplemented with 10% Fetal Bovine Serum, 2 mM L-glutamine, 100 U/mL penicillin and 100 U/mL streptomycin.
    ii. Sub-culture of the cells: When the cells reach about 85% confluency in the flask, the cells need to be sub-cultured for maintenance or to be seeded onto coverslips for an experiment. To sub-culture the cells the following steps are performed in a laminar flow cabinet using sterile equipment. First, discard the media in which the cells are grown. Then wash the T25 flask bottom with 5 mL PBS, and discard the PBS. Next, add 1 mL 0.05% Trypsin EDTA to the T25 flask. The trypsin will detach the cells from the flask bottom and break-down the adherence between cells. Place the flask in the incubator for 3 min or until the cells are floating in the solution. The user can check under brightfield illumination. In the meantime, add 5 mL supplemented DMEM/F-12 to a new T25 flask. Label the flask with date, cell passage, name, and cell line, or as the user sees fit. Once the cells become detached, add 4 mL supplemented DMEM/F-12 media and gently mix by pipetting. For maintenance add 1/10 of the cell suspension (0.5 mL) to the new T25 flask and return to incubator. This will be ready for sub-culturing again in 3 days. To seed cells for immunofluorescence experiment, add a sterile coverslip to the well of a 6-well plate (this is one sample, do as many as required for the desired experiment). Then add 2 mL of supplemented DMEM/F-12 to the well containing the cover slip. Finally, add 500 mL of the cell suspension created above to the well. The cells will be ready for fixation in two days (see step b).
      ***Note:*** The coverslip size and thickness should be chosen such that it best fits the used microscope. We suggest use of N1.5 coverslip, 22 by 22 mm square.
    iii. Freezing and storage of cells. To freeze cells, **first** prepare a T75 flask by adding 15 mL supplemented DMEM/F-12 and 1.5 mL of the cell suspension from step ii. above. Grow the cells for two days. Then, discard the media and wash the flask bottom with 10 mL PBS and discard the PBS. Add 2 mL trypsin-EDTA to the flask and incubate for 2 min or until the cells have detached. Add 8 mL of supplemented DMEM/F-12 media and gently mix by pipetting. Transfer the cell suspension to a 15mL falcon tube and centrifuge for 5 min at 1200 rpm. Discard the supernatant and re-suspend the cell pellet in 10mL freezing medium. The Freezing medium recipe is: 10% DMSO, 17% FBS and 73% non-supplemented DMEM/F-12. Add 1 mL from the cell suspension in freezing media to a cryotube. Place the cryotube in a freezing container (to slow down cooling temperature to around 1°C/min) and place at -80 for about 24 h. Transfer to liquid nitrogen storage on the following day.
  b. Immunofluorescence protocol: the following immunofluorescence protocol has been tested on human hTERT-RPE1 and HeLa K cells and used for development of this methodology Here, the cells are ‘‘fixed’’ by cross-linking the proteins in the sample via a paraformaldehyde-containing solution. The user can also use alternative fixation protocols, as long as the recommended controls in the ‘‘Before you begin’’ section give similar results. The following steps do not require sterile environment.
    i. Prepare PTEMF (fixation solution) - 4% paraformaldehyde, 0.2% Triton X-100, 10mM EGTA (pH = 7), 20mM Pipes (pH = 6.8), 1mM MgCl_2_, and double distilled water. Always prepare fresh on the day of fixation. About 2 mL per sample is sufficient.
      **CRITICAL:** To obtain best fluorescent staining, the user is recommended to be careful to not dry out the cover slip.
      **CRITICAL:** Always wear PPE and use fume cabinet with airflow when working with **PTEMF** – paraformaldehyde is **highly toxic**!
    ii. Gently discard the media with Pasteur pipette. Try not to touch the cells on the coverslip.
      ***Note:*** Tilting the 6-well plate at about 60 degrees from the bench will ease discarding and adding solutions without disturbing the cover slip.
    iii Add PTEMF to the well with a Pasteur pipette such that the PTEMF covers the cover slip with cells, about 2mL. Incubate for 10 min at 20°C–26°C. In the incubation time, prepare 3% Bovine Serum Albumine (BSA) in PBS solution.
      ***Note:*** BSA is very sticky. Add the BSA powder to the PBS and let it dissolve. Do not stir.
      ***Note:*** The 3% BSA in PBS solution can also be prepared before you begin the fixation of the cells. However, it should be prepared fresh on the day when it will be used.
    iv. Washes: Discard the PTEMF in a bottle for toxic waste with a Pasteur Pipette. With a Pasteur pipette add PBS enough to cover the coverslip (about 1.5 mL). Incubate for 5 min at 20°C–26°C. Discard the first PBS wash in the bottle for toxic waste, and add fresh PBS as before. Incubate for 5 min at 20°C–26°C. Discard the PBS in non-toxic waste container. After the first wash, only traces of Paraformaldehyde remain and the following washes are thus non-toxic. Add fresh PBS as before and incubate for 5 min at 20°C–26°C .
      **Pause point:** To pause for up to 24 h, add fresh PBS to the sample, making sure the cover slip is covered, and place the 6-well plate at 4°C.
    v. Blocking: Discard the PBS with a Pasteur pipette. Add 3% BSA in PBS, such that it covers the cover slip (~ 1.5 mL). Incubate for 30 min at 20°C–26°C.
      ***Note:*** Why we do “blocking” - The BSA protein in the blocking solution is “sticky” and binds to the coverslip glass. Therefore, it “blocks” the non-specific binding sites of the antibodies on the glass cover slip, and decreases the extent of non-specific binding by the primary and secondary antibodies.
      ***Note:*** The incubation can be extended up to about 2 h without affecting the sample.
    vi. In the blocking incubation time, prepare the following “antibody incubation chamber”: cut a square of thick filter paper such that it can fit in a 15 cm diameter plastic tissue culture dish. Wet the filter paper with tap water and place it in the 15 cm dish. Cut a piece of parafilm, roughly the size of the filter paper and place on top of the filter paper.
      ***Note:*** the following sub-steps of iii. are to be done immediately before the blocking time is over to prevent degradation of the antibody.
    vii. Dilute the primary antibody in 3% BSA in PBS to the working dilution recommended for immunofluorescence, such that a single final mix containing all primary antibodies is obtained. Vortex the mixture. The working dilutions for commercially available antibodies tested for this protocol are given in the key resources table.
      **CRITICAL:** the stained kinetochore marker needs to (1) be present in interphase cells and (2) stain well in Gaussian spot shape manner, i.e. CenpA or CenpC (avoid use of anti-centromere antibodies, known as CREST antibodies, which often results in a “spread” signal).
    viii. Place 100 μL of the antibody mix in a drop on the parafilm in the “antibody incubation chamber”.
    ix. With forceps carefully take the cover slip out of the 6-well plate, holding it near the edges. Discard the liquid by holding the cover slip vertical and tapping the edge onto a tissue. Place the cover slip on top of liquid drop in the “antibody incubation chamber”. Close the 15 cm dish.
      **CRITICAL:** Make sure the cells on the cover slip are facing down in the “antibody incubation chamber”. In the 6-well plate, the cells are on top of the cover slip.
    x. Incubate for 1 h at 20°C–26°C.
    xi. Cut a piece of parafilm and place it on top of a 6-well plate lid.
    xii. Carefully, take the cover slip with forceps from the “antibody incubation chamber” and discard the liquid by holding the cover slip horizontally and tapping the edge onto a tissue.
    xiii. Place the cover slip with cells facing up onto the parafilm on top of the 6-well plate. Repeat the following procedure 3 times: Add ~1 mL PBS with a Pasteur pipette and incubate for 5 min. Discard the PBS with Pasteur pipette or aspirator set at minimum speed. Do not discard the PBS after the last wash.
    xiv. Wipe the parafilm in the “antibody incubation chamber” with a tissue such that none of the primary antibody mix liquid remains. Dilute the secondary antibodies in 3% BSA in PBS to the same working dilution, creating equal mixture. Vortex the mixture.
    xv. Place a 100 μL of the antibody mix in a drop on the parafilm in the “antibody incubation chamber”.
    xvi. With forceps carefully take the cover slip out of the 6-well plate, holding it near the edges. Discard the liquid by holding the cover slip vertical and tapping the edge onto a tissue. Place the cover slip on top of liquid drop in the “antibody incubation chamber”. Close the 15 cm dish. Cover the dish with foil to prevent bleaching of the fluorescence.
      **CRITICAL:** Make sure the cells on the cover slip are facing down in the “antibody incubation chamber”. On the 6-well plate lid, the cells are on top of the cover slip.
    xvii. Incubate for 30 min at 20°C–26°C.
    xviii. Wash the coverslip by following steps xi to xiii.
    xix. Clean a microscope slide with a piece of tissue and label the slide appropriately.
    xx. Add a drop of VectaShield in the middle of the microscope slide (about 20 μL).
    xxi. With forceps carefully take the cover slip out of the 6-well plate, holding it near the edges.
    xxii. Discard the liquid by holding the cover slip vertical and tapping the edge onto a tissue.
    xxiii. Place the cover slip on top of the VectaShield drop. Do not move the slide after placing to prevent smudging of the cells.
    xxiv. Seal the cover slip to the microscope slide by applying nail varnish to the edges.
    xxv. Cover the slides with opaque box or foil and leave horizontally to dry. Leave for 1–2 h at 20°C–26°C or for about 12 h at 4°C.
    xxvi. Keep the slides in microscope slides box at 4°C when not in use.
  c. Image the ACS: See the before you begin section for details on the choice of imaging set up. This protocol has been tested on spinning disk microscope with pixel size 0.069 nm in the xy plane. Other microscopes with similar resolution, i.e., with similar pixel size and objective can also be used (see steps 1–3). Take a 12 micrometer unit z-stack, at 100 nm z-step intervals (121 images), aiming for one or two interphase cells in the center of the field of view. Choose the z sampling such that it is Nyquist sampling or lower (see steps 1–3). Take a dataset of 10 to 15 images of different cells for a given day of imaging. **For example:** For the development of the protocol, we used a confocal microscope with Nyquist sampling calculated as above to be 135 nm. However, as described above, due to the microscope characteristics we could increase the sampling up to 228 nm without significantly reducing the resolution. For the ACS imaging, we used 100 nm z sampling (oversampling) to ensure we capture significant information for as accurate as possible estimation of the chromatic shift.
    **CRITICAL:** The user is advised to adjust the acquisition settings such that the spot signal-to-noise ratio (SNR) is higher than 2.
    ***Note:*** The z-stack size is chosen to be 12 μm as this is enough to sample a full mitotic human cell. If the user is studying another subject with different depth, the z-stack size is to be optimized accordingly. However, the user should consider that if a much larger object is imaged in depth, the resolution may be affected.
    **CRITICAL:** The ACS dataset is a control for the optical alignment of the microscope. The microscope optical alignment could change over time. Therefore, the ACS dataset should be imaged on the day of sample imaging.
    ***Note:*** For ACS, interphase cells are used instead of fluorescent beads as they better represent the diffraction in a cellular environment. Therefore, initial microscope alignment can be performed by beads but for detection of day-to-day alignment variations and correction in the software, the described ACS slide is recommended. Here, interphase cells are used instead of mitotic cells to facilitate data acquisition and analysis (only ~2–10% of hTERT RPE1 cells in culture are in mitosis).
    **CRITICAL:** The KiDv1.0.1 software supports only three fluorescent channels and any additional channels are not seen in the analysis. If the user would like to acquire images in channels that would not be used in analysis (such as DAPI), the user should acquire in those channels last, i.e. the first three acquired channels should be the channels used for analysis if more than three channels are acquired.
  d. Export all images as ome.tif or .r3d files.
    ***Note:*** If the microscope does not support one of those formats (such as 3i systems which use SlideBook), open the image in Fiji and save it as ome.tif file.
    ***Note:*** The user is advised to include consecutive numbering in the image names for the acquired images in a sample. Image stack numbering is not required by the software but will facilitate relating the analysis in KiDv1.0.1 to the original file number. **For example:** Initials_date_chromaticshift_protein_colors_number: TG_01062021_chromaticshift_CenpA_488561647_01

2. Sample preparation.
  a. This protocol is developed for human adherent cells. An example of how to grow and maintain human non-transformed hTERT RPE1 cells is provided in 1 a. To prepare hTERT RPE1 mitotic cells for immunofluorescence follow the instructions in step 1 a ii.
  b. Immunofluorescence: the fixation and immunostaining are performed as in step 1 b, except the following considerations:
    i. The user is not limited to antibodies and can also use fluorescently tagged proteins for labeling of kinetochore markers.
      **CRITICAL:** If fluorescent proteins are used for the labeling, always cover the sample with foil in the incubation times to prevent photo bleaching.
    ii. When adding and discarding solutions, the user is advised to do it gently, especially before the cells are fixed in PTEMF. This is specifically necessary for mitotic sample preparations to prevent detachment of the mitotic cells and their loss.
      ***Note:*** When hTERT RPE1 cells enter mitosis, they round up and are not that adherent to the dish.
    iii. Primary antibodies – the user is recommended to use up to three primary antibodies as the software can process up to three kinetochore markers labeled in different fluorescent channels.
      **CRITICAL:** Use antibodies raised in different species so they can be recognized by different secondary antibodies and this way labeled in different colors.
    iv. Secondary antibodies: for the development of this protocol we used the Alexa fluorescently conjugated antibodies specified in key resources table. The user is recommended to use these antibodies where fitting the primary antibody species as we have shown these antibodies to work with the above protocol.
      **CRITICAL:** Use secondary antibodies raised against the primary antibody species.
  c. Image the sample with the same microscope set up as the ACS slide and as described in the “Before you begin” section.
    **CRITICAL:** We advise the user to optimize the image acquisition such that the SNR is between 2 and 3. Images with higher SNR can also be used. Directions on troubleshooting and optimization in the cases when not sufficient spots are detected are available in the troubleshooting section and later in the protocol.
    ***Note:*** In our experience, dataset of 17 to 24 cells per experiment is typically sufficient to produce a dataset of 300-500 kinetochores, depending on the quality of the signal.
    **CRITICAL:** We do not recommend oversampling in the z direction when imaging the sample. As discussed before, for our imaging system the Nyquist sampling is 135 nm and can be increased to 228 without significantly decreasing the resolution. Therefore, for sample imaging we used 200 nm sampling in z.
    **CRITICAL:** The KiDv1.0.1 software supports only three fluorescent channels and any additional channels are not seen in the analysis. If the user would like to acquire images in channels that would not be used in the analysis (such as DAPI), the user should acquire in those channels last, i.e. the first three acquired channels should be the channels used for analysis if more than three channels are used.
  d. Export all images as ome.tif or .r3d files.
    ***Note:*** The user is advised to include consecutive numbering in the image names for the acquired images in a sample. Image stack numbering is not required by the software but will facilitate relating the analysis number in KiDv1.0.1 to the original file number.

### Entering raw data in KiDv1.0.1 and setting up the analysis

**Timing: 2–6 h for step 3**

**Timing: 2–6 h for step 4**

This section provides instructions how to (1) use the KiDv1.0.1 Graphical User Interface (GUI), (2) input raw data and specifications and (3) select the analysis to perform. All of this information is saved in a jobset file.

Troubleshooting 1 and 2

**CRITICAL:** Every time the user closes and opens MATLAB, the user should add the “KiDv1.0.1” and “BEDCA” folders to the MATLAB path, see step 7b from the "Before you begin" section.

**Figure 3:**
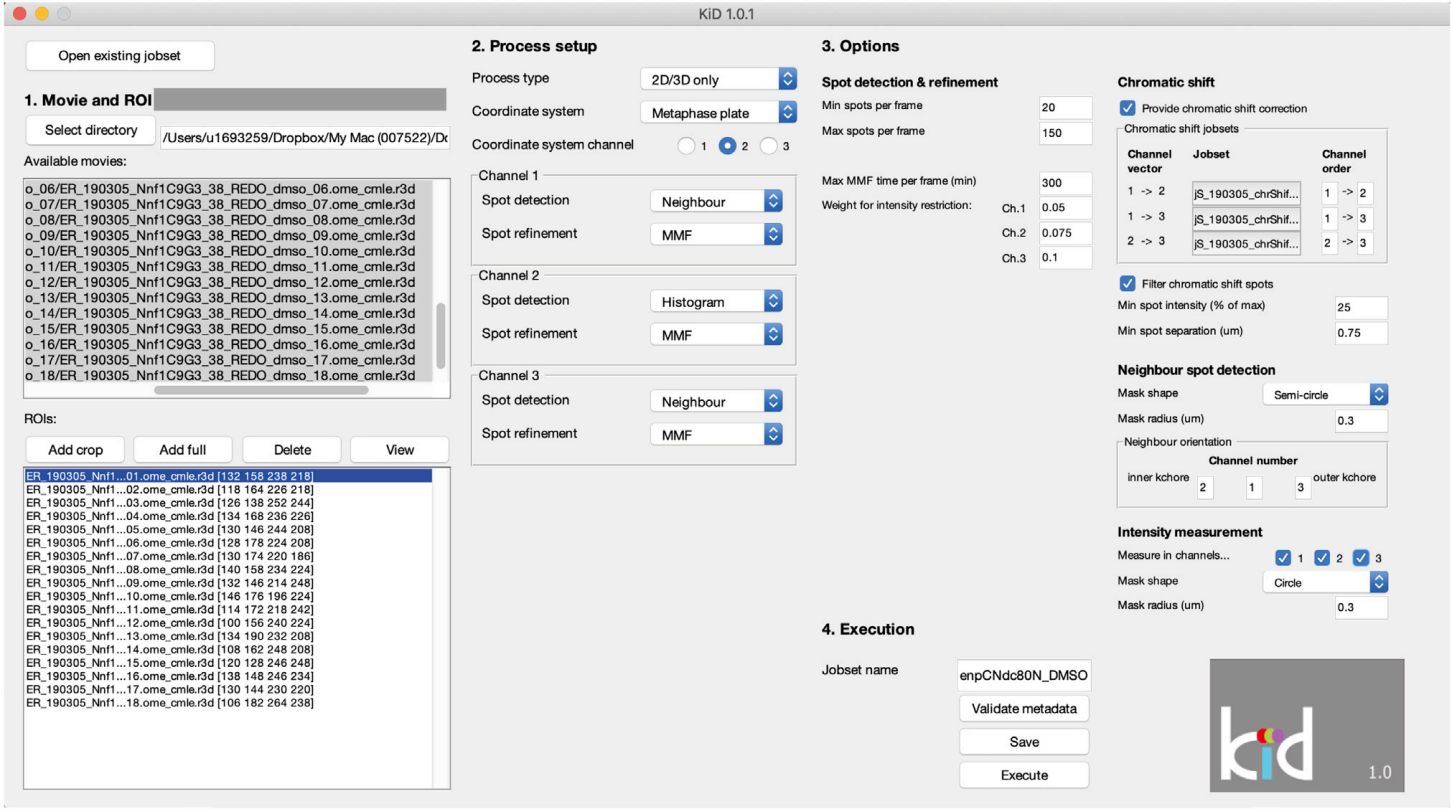
Kinetochore Delta (KiDv1.0.1) software graphic user interphase (GUI) for data analysis set up The parameters of data anlysis are set up and saved in a jobset as described in steps 3 and 4. The figure displays the jobset analysis set up of the provided Nnf1-CenpC-Ndc80N sample dataset as described in step 4. (See also Figures S1 and S2)
    **For example:** jS_exp1_13042020_ACS
    ***Note:*** The user is advised to use informative naming. Here, we suggest a naming system that has been used for the set of experiments in Roscioli *et al.* However, the user can develop their own naming system as appropriate.
  b. Movie and ROI selection
    i. Click on **Select directory***.* Select the folder that contains the ACS images and click on **Select Folder***.* You can now see the path of the selected folder on the right of the **Select directory** button, and all of the files in the selected folder in the Available movies window.
      ***Optional:*** If the user has already saved a jobset (at the end of step 3) and wishes to edit it, the user can click on **Open existing jobset**.
      ***Note:*** Folders opened through the KiD1.0.1 GUI do not need to be added to the MATLAB path as they do not contain MATLAB code.
    ii. In the Available movies window, select the image stacks that are to be used for ACS correction by clicking with left mouse button on the stack – it will become highlighted in blue.
      ***Note:*** You can select multiple image stacks at once by selecting the first image, holding **Shift** and selecting the last image, or by selecting one image and holding **Command** to select the rest.
    iii. Click on **Add full***.* This will enter the full image size for each selected image stack to the bottom of the list in the ROIs window. The image stacks used for ACS correction contain interphase cells and thus cropping is not required.
      ***Note:*** If the user has made a mistake, an ROI can be deleted by selecting it with left mouse button and clicking **Delete***.* The ROI can also be viewed in similar manner using the **View** button.
    iv. Repeat steps ii. and iii. until all ACS images are added to the ROIs window.
  c. Process setup
    i. In the Process type drop down menu select Chromatic Shift.
    ii. In the coordinate system (CS) channel pick the fluorophore channel with the best signal. Here O1 is the first, O2 is the second, and O3 is the third acquired channel.
      **CRITICAL:** To choose the best channel, the user is recommended to first choose the channel with best signal-to-noise ratio (ideally greater than 2) and run all of the sub-steps in step 3. After the job has been processed in step 3. the user is recommended to check the number of detected spots per image stack (see step 3 f.). Since healthy human cells have 46 kinetochores in interphase, we expect to be able to detect close to this number of spots. If the average number of detected spots per cell is 23 or more (50% of total), the user can proceed using this channel as the coordinate system channel in further experiments. If the detection yields less than 50% of the expected number of spots per cell then the channel is sub-optimal. The user is therefore recommended to re-load the jobset (as in step 3 b i. Optional), and amend it such that another channel is the coordinate system channel. Then again check the total yield of the detection. If all of the channels are sub-optimal, the user is suggested to consult with the troubleshooting provided. **For example:** for ACS correction slide, cells are stained with anti-CenpA primary antibody labeled with a mixture of A488-, A561- and A647-conjugated secondary antibodies. Here, running step 3 with Channel2 (CenpC-A561) as coordinate system channel yields 775 spots after processing of 10 ACS image stacks. Therefore, Channel2 (CenpC-A561) is selected for coordinate system channel by selecting O2.
    iii. The detection method for the CS channel is automatically set to Histogram and the refinement to MMF. Using these parameters, KiDv1.0.1 will first detect spots in the CS channel via unimodal histogram thresholding. In this method, the histogram of all signal intensities is used to determine the threshold that discriminates between signal and background noise. Next, KiDv1.0.1 models the spots based on the default PSF and detects the likely candidates. Finally, the detected spots are refined using Gaussian mixture model fitting (MMF) and their centers are detected. The MMF function checks how many Gaussian spots best explain the detected signal and improves kinetochore detection in cases of two nearby or overlapping kinetochore signals.
      ***Note:*** The options in the KiDv1.0.1 GUI is denoted here with semicolon before the option such as :Histogram.
    iv. Set the detection in the non-coordinate system (NCS) channels with kinetochore marker to :Neighbor and the refinement to :MMF. The NCS channels are all acquired channels with kinetochore marker except the channel selected as CS channel in step ii. The option :Neighbor allows the software to search for the spot signal in a NCS channel within a :Circle or :Semi-circle (see mask options, step d ii.) centered at the corresponding spot coordinates in the CS channel.
      ***Note:*** The :Semi-circle mask is used only when there is a metaphase plate such that the :Semi-circle can be oriented relative to the metaphase plate. In all other cases, the :Circle option is used. This protocol uses interphase cells for ACS correction and in interphase cells there is no metaphase plate. Therefore, for ACS processing the :Circle option is the only one that can be used. The user will encounter the :Semi-circle mask when processing the sample. **For example:** for CenpA-A488-A561-A647, the CS Channel is Channel2 (CenpA-A647). Therefore, the NCS channels are Channel1 (CenpA-A488) and Channel3 (CenpA-A647).
      ***Note:*** Through this setup the best signal is used for primary spot detection, while the detection in the other channels is facilitated by searching only in a mask with a set shape and radius from the primary identified spot.
  d. Options: Spot Detection and Refinement.
    i. Min spots per frame :20 denotes that KiDv1.0.1 will aim to use a :Histogram threshold such that a minimum of :20 spots are detected in the image stack.
      ***Note:*** Healthy human cells in mitosis have 92 kinetochores. Therefore, :20 Min spots per frame is a low threshold. However, we have found that using a more accurate range does not significantly improve the detection. Therefore, a more permissive range is left such that if treatments affect the cell kinetochore number, the detection can be comparable to the controls. If the user utilizes a treatment or model organism where less than 20 kinetochores are expected, the user is recommended to adjust the default setting appropriately. **For example:** Budding yeast have 16 kinetochores in mitosis and thus 20 is not a reasonable number for that system. In this case a min of :0 spots will be more befitting.
      ***Note:*** Similar as for the Min spots per frame, the Max spots per frame threshold are not restrictive for normal human cells. However, if the user utilizes a treatment (or cell type/organism) where more than 150 kinetochores are expected, the user is recommended to adjust the default value. **For example:** Drugs can be used to create tetraploid human cells. Tetraploid cells will have 184 kinetochores. Therefore, in such case the user can set a Max spots per frame threshold of :200 such that it is appropriate for this condition.
    ii. Max MMF time per frame (min) :300 denotes that the MMF algorithm in KiDv1.0.1 will try to refine the spots for a maximum of 300 min.
    iii. Weight for intensity restriction – signal weight is applied as the signal in the 488 nm channel is typically the strongest, followed by the 561 nm channel, and the 647 nm channel which is the weakest.
  e. Options: Neighbor spot detection
    i. Mask shape – KiDv1.0.1 starts from the kinetochore center location in the CS channel and searches in :Circle (only mask available for ACS processing) to find the spots in the :Neighbor system channel.
    ii. Mask radius (μm) – defines the radius of the mask in which KiDv1.0.1 will search for the neighbor spot.
      ***Note:*** The default mask radius is :0.3 μm (300 nm). This default reflects the size of the human kinetochore which is approximately 300 nm. With size of 300 nm of the kinetochore as a whole we do not expect the individual components to be more than 300 nm apart. However, if the user is assaying another structure, the mask radius is to be adjusted such that the radius covers the maximum expected distance between the coordinate system channel component and the component in the Neighbor system channel.
    iii. Channel number: type the order of channels from inner toward the outer kinetochore components based on the most likely linear arrangement of the kinetochore markers used in the experiment. If only two channels are detected, one box will remain grayed out.
      ***Note:*** For ACS correction, the same marker is stained in three colors so the order can be left as default.
  f. Intensity measurements. The user can choose to make intensity measurements in any of the channels by clicking on the box next to the channel number.
    ***Note:*** Intensity measurements are not typically useful for the ACS Correction.
    i. Mask shape – KiDv1.0.1 starts from the kinetochore center location in the CS channel and measures the intensity in a :Circle (only mask available for ACS processing) in the specified channels for intensity measurements.
    ii. Mask radius (μm) – defines the radius of the mask in which KiDv1.0.1 will measure intensity.
      ***Note:*** The default mask radius is :0.3 μm (300 nm). This default reflects the size of the human kinetochore which is approximately 300 nm. With size of 300 nm of the kinetochore as a whole we do not expect the individual components to be more than 300 nm apart, or larger than 300 nm. However, if the user is assaying another structure, the mask radius should be adjusted appropriately.
  g. Execution
    i. Type or paste the jobset name defined in step 3a in the Jobset name field.
    ii. Click on Validate metadata and check that the metadata is read in correctly.
    iii. Click Execute
      ***Note:*** by default MATLAB will hint all functions and variables that start with the typed phrase if the user presses tab. MATLAB will display the associated with a function help, if the user types in the Command window help followed by name of the function.
  h. Detection Quality. Once tracking is complete, KiDv1.0.1 will display in the Command window ‘‘Tracking complete’’. Scroll up in the Command window and check how many spots have been detected. If the average number of detected spots per cell is 23 or more (50% of total, based on 46 kinetochores in healthy human interphase cell), the user can proceed to step 4. If the detection of spots did not meet the above criteria see the associated troubleshooting.

3. Processing the ACS data troubleshooting 1 and 2
  In this step the user will input the ACS images in KiDv1.0.1 in the form of a jobset (jS) file that contains information about the data, analysis set up, and once it is processed at the end of step 3, also the initial raw data analysis. For more information about the ACS correction see step 1.
  ***Note:*** at the end of step 3, the jobset analysis will be automatically saved in the folder with the raw data.
  a. Creating a jobset(jS) file. In the Command window type jS_Name = kitGUI, where jS_Name is the name under which the analysis performed in this section will be saved. This command will open a GUI where the user sets up the ACS analysis for the day of imaging (see Figure 3 and Figures S1 and S2).
    ***Note:*** We suggest the name to be in the following format: jS_expN_DateofImaging_ACS

4. Processing the Sample data Troubleshooting 1 and 2
  In this step the user will input the raw Sample data in KiDv1.0.1 by creating a jobset (jS) file that contains information about the data, analysis set up, and once it is processed at the end of step 4, also the initial raw data analysis.
  ***Note:*** at the end of step 4, the jobset analysis will be automatically saved in the folder with the raw data.
  a. In the Command window type jS_Name = kitGUI, where jS_Name is the name under which the analysis performed in this step will be saved. This will open a GUI where the user can set up the sample analysis for the day of imaging (see Figure 3 and Figures S1 and S2).
    ***Note:*** The Graphic user interface does not display fully on Windows OS. If the user is running KiDv1.0.1 on Windows OS, the user is recommended to navigate in the GUI with the help of Figure 3 and Figures S1 and S2.
    ***Note:*** We suggest the name to be in the following format: jS_expN_DateOfImaging_ProteinsInChannelOrder_Treatment. **For example:** in the provided experiment, hTERT RPE1 cell line is stained with anti-Nnf1, anti-CenpC and anti-Ndc80 N-terminus (Ndc80(N), 9G3 antibody) primary antibodies which are then labeled with Alexa 488-, Alexa 561- and Alexa 647-conjugated secondary antibodies, respectively. The respective jobset can be named: jS_exp1_13042020_Nnf1CenpCNdc80N_DMSO and will identify Nnf1, CenpC and Ndc80(N) in the 488nm, 561nm and 647nm fluorescent channels, respectively.
  b. Movie and ROI selection
    i. Click on **Select directory***.* Select the folder that contains the Sample images and click on **Select Folder***.* The user can now see the path of the selected folder on the right of the **Select directory** button, and all of the files within the selected folder in the Available movies window (as in 3b)
      ***Optional:*** If the user has already saved a jobset (at the end of step 3 or 4) and wishes to edit it, the user can click on **Open existing jobset** and load it. If ACS jobsets have been loaded, KiDv1.0.1 will open a browser window where the user is to select the ACS jobset.
    ii. Select all of the movies to be used for Sample analysis as in 3b ii.
      **CRITICAL:** Make sure that the cells are added in the order of the raw image numbering, i.e., raw cell 1 is added first and so on. This way the movie numbering in the analysis will correspond to the image stack numbering of the raw data.
    iii. Click on **Add Crop***.* To facilitate spot tracking of the sample, it is advisable to use only the part of the image where the mitotic cell is located. This button will open a figure with a maximum Z projection of the image stack.
      ***Note:*** If the user has used field of view size such that only the cell of interest and a little bit of background is captured (such as 256x256 pixels), the user can add the full image as described for ACS images in step 3 b. and continue to step 4 c.
    iv. Click on **Add ROI** and make a rectangle around the mitotic cell including some background. Avoid high background signals and other nearby cells.
    v. Double click in the rectangle to confirm it. Click **Finish**.
      ***Note:*** The user can also add a second ROI in one image by clicking **Add ROI** again. In that case, make a note of the image stack with more than one ROI as now the number of ROIs will be more than raw image stacks and thus the numbering in the analysis and raw data will differ.
      ***Note:*** After step v., the ROI will appear at the bottom of the list in the ROIs window. Here, if the user has made a mistake, an ROI can be deleted by selecting it with left mouse button and clicking **Delete***.* The ROI can also be viewed in similar manner using the **View** button.
    vi. Repeat steps ii. to v. until all cells are added to the ROIs list.
  c. Process setup
    i. Process type. Select 2D/3D only.
    ii. Coordinate system. Select :Metaphase plate if the kinetochores are aligned in a metaphase plate. Select :Center of mass if metaphase plate is not clearly present (e.g., prometaphase cells and cells treated with nocodazole).
      ***Note:*** The software does not currently support analysis of anaphase cells.
    iii. Coordinate system channel. Choose a coordinate system (CS) channel based on the following criteria: (1) the protein is a robust kinetochore marker, i.e., does not get loaded/unloaded dynamically, (2) has a clear kinetochore-localized signal, (3) if relevant, can serve as a reference in multiple experiments.
      **For example:** for Nnf1-A488, CenpC-A561, Ndc80(N)-A647, the best marker to use as CS channel is Channel2 (CenpC-A561) as the antibody is raised in guinea pig (uncommon host) and can serve as reference for multiple experiments.
    iv. Set the detection in the non-coordinate system (NCS) channels to :Neighbor and the refinement to :MMF (see steps 3c iii. and iv. for description of these options). NCS channels are all acquired channels with a kinetochore marker **except** the channel selected as CS channel in step iii.
    v. Leave the detection to :None and the refinement to :None in channels with no staining or no kinetochore marker stained.
      **For example:** for Nnf1-A488, CenpC-A561, Tubulin-A647: CenpC produces robust kinetochore staining and is a a good reference marker as the antibody is raised in uncommon host (Guinea Pig). Therefore, Channel2 is set as the CS channel. Nnf1 is a kinetochore marker and Channel2 is the CS channel, therefore, Channel1 is set as NCS channel. Tubulin is not a kinetochore marker and, therefore, no detection is set in Channel3.
  d. Options: Spot detection and refinement – do not change these options for human cells unless there is an issue. See 3d for explanation of the options.
  e. Options: Chromatic shift
    i. Check the box next to Provide chromatic shift correction. Next, the user loads the ACS jobset created in step 3.
    ii. To run detection in two channels, click on the field under Jobset positioned on the right of the used channel pair (i.e., channel vector 1-2).
    iii. To run detection in three channels, load the ACS jobset into all jobset fields (1-2; 1-3; 2-3). **For example:** for Nnf1-A488, CenpC-A561, Ndc80(N)-A647, spot detection is set for all channels and the ACS jobset is loaded into all jobset fields.
      ***Note:*** ACS dataset is required for each day of sample imaging.
    iv. Check the box for Filter chromatic shift spots.
    v. Min spot intensity (% of max) – enter :25 to consider only chromatic shift spots that have intensity which is 25% or higher of the maximum image intensity for chromatic shift correction. Using this value means that very weak intensity spots will be discarded.
    vi. Min spot separation – enter :0.75 to discard ACS spots that are closer than 0.75 μm. This parameter filters background and overlapping spots based on the fact that the kinetochore sister-sister pair distance at rest (no force applied) is 0.75 μm.
  f. Options: Neighbor spot detection
    i. Mask shape – KiDv1.0.1 starts from the kinetochore center location in the CS channel and searches in a :Semi-Circle or :Circle area to find the spots in the Neighbor system channels. The :Semi-Circle is used when :Metaphase plate option is given in step 4c ii to orientate the search area relative to the metaphase plate. :Circle is used in all other situations.
    ii. Mask radius (μm) – defines the radius of the mask in which KiDv1.0.1 will search for the neighbor spot.
      ***Note:*** The default mask radius is :0.3 μm (300 nm), see 3 e ii for more information.
    iii. Channel number: type the order of channels from inner toward the outer kinetochore components based on the most likely linear arrangement of the kinetochore markers used in the experiment. If only two channels are detected, one box will be grayed out. **For example:** for Nnf1-A488, CenpC-A561, Ndc80(N)-A647, the channel order will be 2 1 3.
  g. Options: Intensity measurements. The user can choose to make intensity measurements in any of the channels by clicking on the box next to the channel number.
    ***Note:*** Check all the boxes if in the future intensity measurement may become useful as these are quick to run and if afterwards such analysis is required, the full jobset will need to be re-analyzed.
    i. Mask shape – KiDv1.0.1 starts from the kinetochore center location in the CS channel and measures the intensity in a :Circle or in a :Semi-Circle in the specified channels for intensity measurements. The :Semi-Circle is oriented as described for Neighbor spot detection above (see step 4 f.) and can be used only when the Process type is set to :Metaphase plate. For the work in Roscioli et al., 2020, all intensities were measured using the :Circle option as it gives better intensity coverage.
    ii. Mask radius (μm) – defines the radius of the mask in which KiDv1.0.1 will measure intensity.
      ***Note:*** The default mask radius is :0.3 μm (300 nm), see 3 f ii. for more information.
      **CRITICAL:** This approach to intensity measurements is to be used for kinetochore-localized markers **only**.
  h. Execution
    i. Put the Jobset_Name chosen in step 4a. in the Jobset name field.
      **For example:**jS_exp1_13042020_Nnf1CenpCNdc80N_DMSO
    ii. Click on Validate metadata and check that the metadata is read in correctly.
    iii. Click Execute
      ***Note:*** The jobset is now saved in the folder where the source images are. It will also appear in the Workspace window meaning that it is accessible from MATLAB at the moment.
      ***Note:*** The user can also click Save to save the jobset and execute it at another time after re-loading it into the GUI as described in step 4 b i.
      **CRITICAL:** if the user closes and then re-opens MATLAB, the jobset will not appear in the Workspace window, i.e. the user will need to load it into MATLAB for further analysis. To do that, type jS_Name = kitLoadJobset. Navigate to the raw data window and select the jobset saved with name chosen in 4 a. Click Open. The jobset is now loaded and you can see it in your Workspace window. **For example:** jS_exp1_13042020_Nnf1CenpCNdc80N_DMSO = kitLoadJobset.
  i. Detection Quality. Once tracking is complete, KiDv1.0.1 will display in the Command window ‘‘Tracking complete’’. Scroll up in the Command window and check how many spots have been detected. If the average number of detected spots per cell is 46 or more (50% of total, based on 92 kinetochores in healthy human metaphase cell), the user can proceed to step 5 for Delta distance analysis. If the detection of spots did not meet the above criteria see the associated troubleshooting.

***Note:*** The statistics of spot detection are also saved in a .txt file in the raw data folder.

### Quality control of detected kinetochore spots and cells

**Timing: 2–8 h for step 5**

**Timing: 4–16 h for step 6**

**Timing: 4–16 h for step 7**

**Timing: 4–16 h for step 8**

Cell selection, sister kinetochore pairing and quality control of detected spots are essential steps to improve the calculation of intra-kinetochore distances. This section provides instructions to select cells, to pair sister kinetochores and to run spot quality control in experiments where two/three kinetochore markers are stained. This section also includes instructions on kinetochore marker spot categorization by eye.

5. Cell selection, manual kinetochore sister pairing and spot quality control in the CS channel troubleshooting 3. **For example:** the staining is Nnf1-A488, CenpC-A561, Ndc80(N)-A647 and the CS channel for primary spot detection is Channel2 (CenpC-A561). The goal is to select the ‘‘good’’ cells for the analysis, pair the detected sister kinetochores and select the good quality spots in the CS channel. ‘‘Good’’ cells are considered those that efficiently respond to a treatment (e.g., cells with no microtubule stubs upon nocodazole treatment) and cells that satisfy a criterium of selection based on the goal of the experiment (e.g., cells with all kinetochores aligned in a metaphase plate when the goal is to study the kinetochore architecture in metaphase)."
  **CRITICAL:** If the user has closed MATLAB and re-opened it after step 4, the user should load the jobset analysis from step 4 as described in step 4 e iii.
  ***Note:*** The kinetochore sister pairing is automatically saved in the jobset during the analysis.
  ***Note:*** The user can stop the pairing at any point by simultaneously pressing on the keyboard **Command** and **C**. The user can then continue the analysis from the next cell by using the ‘Subset’ option (see a iii. below).
  ***Note:*** The user can redo the pairing for one or more cells by using the ‘Subset’ option and indicating those cells. KiDv1.0.1 will display the previous pairing, and any subsequent changes will overwrite the existing pairing and will be automatically saved.
  a. In the Command window type: dublManualPairSisters(jS_Name, options), where in brackets is the jobset name chosen in 4 a. and the options are described next. See step a iv. for example of the function with options.
    i. ‘imageChans’, vector – here the vector contains the channel(s) that KiDv1.0.1 will display for cell inspection and kinetochore pairing. The user should plot the CS channel for pairing. If useful, the user can plot other channels in addition. **For example:** ‘imageChans’, [2] will display only Channel2; ‘imageChans’, [1 2 3] will display the markers acquired in Channel1, Channel2 and Channel3.
    ii. ‘plotChan’, number – the number is the CS channel in which the kinetochore spot centers will be plotted for assessment of the spot quality and pairing. **For example:** if the CS channel is Channel2 (CenpC-A561), this will be given as ‘plotChan’, 2
    iii. ‘Subset’, vector – use this option to do or repeat cell selection on a subset of cells specified in the vector. **For example:** ‘Subset’, [5 6 7 8] will prompt cell selection and pairing only for cells 5, 6, 7 and 8.
    iv. ‘coordChans’, vector – vector is the vector of channels for which pairing will be created. The user should input as vector, the numbers of the CS and all NCS channels. For example: in analysis of cells stained for Nnf1 in 488 nm, CenpC in 561 nm, and Ndc80(N) in 647 nm channel, the vector will be [1 2 3], as all channels are CS or NCS channels.
      ***Note:*** In MATLAB typing [5 6 7 8] is the same as typing [5:8]
    v. **Example:**dublManualPairSisters(jS_exp1_13042020_Nnf1CenpCNdc80N_DMSO, ‘imageChans’, [1 2 3], ‘plotChan’, 2) will display Nnf1-A488, CenpC-A561 and Ndc80(N)-A647 and the spot centers will be plotted for the CenpC-A561 channel.
  b. Cell quality control: Window with a maximum projection of the first cell will appear after step a. To accept the cell in the analysis press y (keyboard). To discard the cell press n (keyboard). See Figure 4.
    **CRITICAL:** Make a note of the accepted and discarded cells. KiDv1.0.1 will display in the Command window in which cell the user is pairing the kinetochores at the moment. Make sure to check the cell number as KiDv1.0.1 will skip any cells where no kinetochores have been detected.
    ***Note:*** in this stage, the user performs a quality control on the acquired cells.
    **CRITICAL:** If the user presses by mistake a keyboard key other than **y** or **n**, KiDv1.0.1 can automatically accept it as **y** and continue the analysis. If the user wishes to discard the cell instead, the function can be terminated by simultaneously pressing on the keyboard **Command and C**. In that case any previous analysis will be saved and the user can continue from that cell by using the ‘Subset’ option (see a.). **For example:** if the mistake happened at cell 3, use the ‘Subset’ option to avoid repeating the analysis for cells 1 and 2.

**Figure 4:**
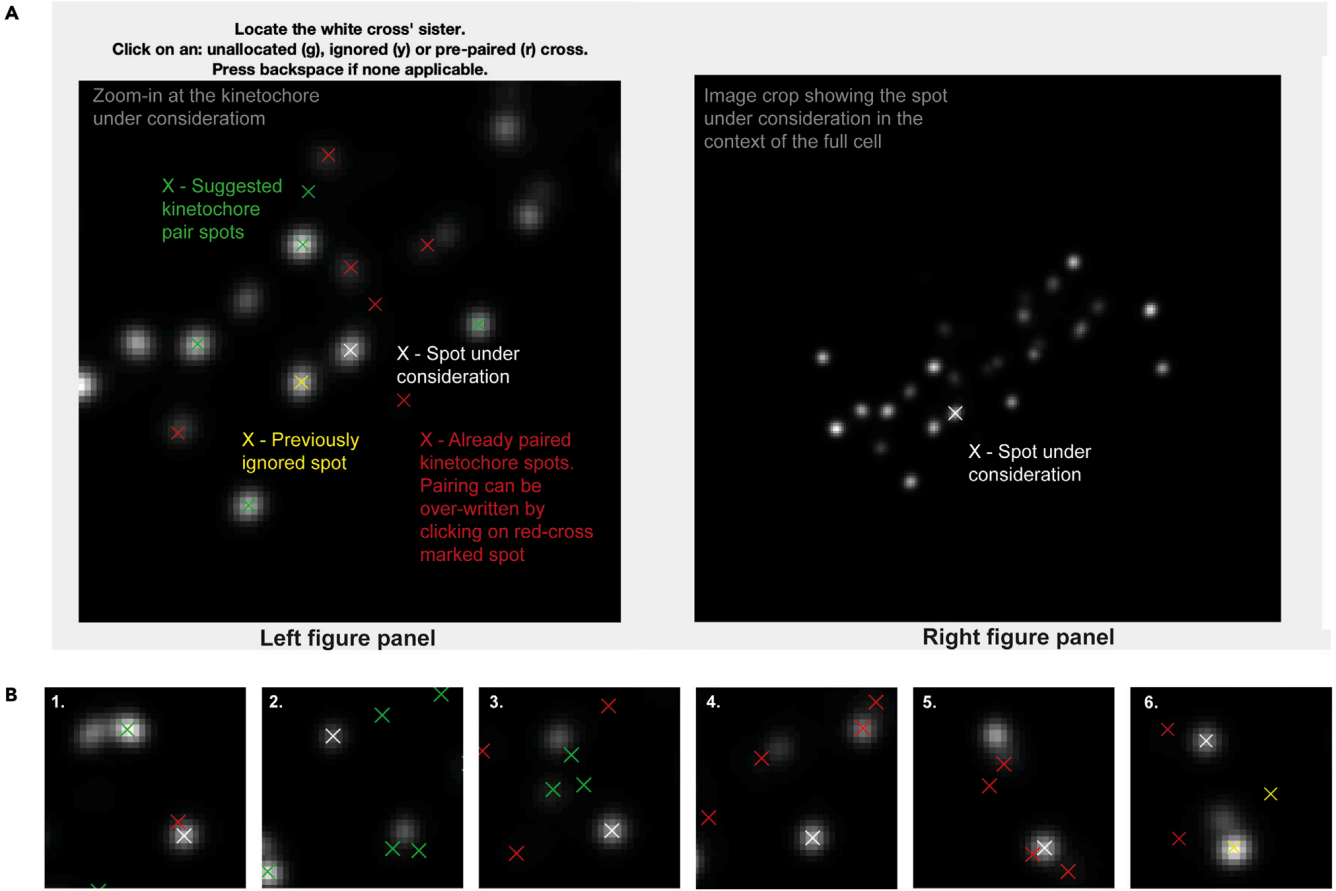
Kinetochore sister-sister pairing and spot quality control in the Coordinate system channel (CSC) (A) Kinetochore sister-sister pairing. Left: zoom-in at the kinetochore spot under consideration (white cross) and spots within two microns that are suggested for pairing (green cross), already paired (red cross) or previously ignored for pairing (yellow cross). The image is projection of 5 z-slices around the spot under consideration. Right: image region of interest as set by the user in step 4, projection of 5 z-slices around the spot under consideration. (B) Examples of kinetochores that should be ignored in the analysis due to spot distortion in the kinetochore under consideration or its kinetochore sister pair (2 and 4), poor detection of the kinetochore spot center (1), and no sister kinetochore spot detection (3, 5 and 6).
  c. Spot quality control and kinetochore sister pairing in the CS channel. Once a cell is accepted, a figure will appear (see Figure 4). The right side shows the full cell in xy, and projection of 5 slices in z. The 5 z-slices are chosen such that the middle slice contains the coordinates of the spot under consideration, and the top two and bottom two are the immediately adjacent z-slices. The left side shows a zoom-in of the spot under consideration (white cross in xy, and the same projection of slices in z. In the zoomed-in image, the green crosses mark suggested spots within 2.5 mm radius from the kinetochore spot under consideration, the yellow crosses mark spots within 2.5 mm radius that have been previously ignored by the user, and the red crosses mark spots within 2.5 mm radius that have been previously paired by the user.
    ***Note:*** KiDv1.0.1 displays the full cell in xy in the right panel to facilitate the user in the choice of the correct kinetochore sister pairing. If required, more advice on sister-pairing is notes.
    ***Note:*** The position of the mouse cursor is visualized as a blue cross spanning the full image.
    ***Note:*** When pairing sister-sister kinetochores, if there is a metaphase plate the user is suggested to: (1) in the right figure panel, identify the direction of the metaphase plate if there is such, i.e. the direction of the plane on which the kinetochore pairs are aligned, (2) in the left figure panel, identify the kinetochore pair such that the K-K axis would lie roughly perpendicular to the metaphase plate, and (3) follow one of the actions below the notes.
    ***Note:*** When pairing sister-sister kinetochores, if there is no metaphase plate present the user is suggested to: (1) in the left figure panel, observe the kinetochore spots close to the one to be paired, (2) in the left figure panel, identify one or more best candidates, (3) see if there is a more suitable pair for some of the candidates in the case where the sister pair is not imminently clear (if the user is really doubtful on which is the correct pair, the user should discard the spot), and (4) follow one of the actions below the note.
    i. if none of the suggested kinetochore spots (green crosses) is the sister pair, press **Space** to exclude the spot.
    ii. if the kinetochore spot under consideration (white cross) or the suggested kinetochore spots (green crosses) are distorted or the center of the spots is not detected correctly, press **Space** to exclude the spot.
    iii. if both the kinetochore spot under consideration (white cross) and the sister kinetochore spot identified by the user are correctly recognized and not distorted, press on the respective green cross to pair the two kinetochore spots.
      ***Note:*** Any kinetochore spots previously discarded will appear marked with a yellow cross.
      ***Note:*** To aid visualization, KiDv1.0.1 auto-scales the intensity of the images to the zoomed-in region.
  d. Repeat steps b. and c. for all cells. This will conclude cell selection, manual sister pairing and kinetochore spot quality control in the CS channel.
  e. Proceed to: step 6 for spot quality control in the NCS channels for Delta measurements or perform step 6 a and b and proceed to step 11 for intensity measurements.
6. Kinetochore spot quality control in one NCS channel for two/three-color Delta measurements. This section illustrates the steps to discard ‘‘bad’’ spots acquired in one NCS channel when at least two kinetochore markers are stained. Spots with non-Gaussian shape and spots with wrong center detection are classified as ‘‘bad’’ spots. **For example:** the staining is Nnf1-A488, CenpC-A561, Ndc80(N)-A647 and the goal of the experiment is tomeasure the intra-kinetochore distance between CenpC-A561 and Ndc80(N)-647. The CS channel is Channel2 (CenpC-A561) and is used for primary spot detection. Channel3 (Ndc80(N)-647) is one NCS channel to be explored for spot quality.
  a. Create a movieStructure (mS). The purpose of this structure is to extract the analysis of the image stacks (movies) completed so far and saved in the jobset. The mS structure will be used as input for further analysis. To do so, in the Command window type: mS_Name = kitLoadAllJobs(jS_Name), where on the left is the name of the mS structure and in brackets is the name of the jobset as given in step 4a. This will load all of the analysis from the jobset into the movie structure.
    ***Note:*** we suggest the mS structure to be named as the jobset**except** starting with mS: mS_expN_DateOfImaging_ProteinsInChannelOrder_Treatment. **For example:**mS_exp1_13042020_Nnf1CenpCNdc80N_DMSO.
  b. Save the movieStructure. In the Workspace window select the mS structure created in a. and click with right mouse button on it, then click with left mouse button on **Save as.** A window will pop-up. In the “Save as” field type the name of the mS structure as defined in a. Then navigate to the folder with raw data and press **Save.** Now the mS structure is saved in the folder with raw data.
    ***Note:*** After closing and re-opening MATLAB the user can load the saved mS structure by dragging it from the folder where it is saved into the Workspace window.
  c. Create a vector denoting the cells that have been selected for analysis.
    Use the list of selected cells made in step 5b and type in the Command window: SelectedCells_Name = [ index number of cells that are accepted ], where on the left is the name of the vector and on the right in square brackets are the numbers of the selected cells.
    ***Note:*** We suggest to name the vector as the jobset**except** starting with SelectedCells: SelectedCells_expN_DateOfImaging_ProteinsInChannelOrder_Treatment**For example:**SelectedCells_exp1_13042020_Nnf1CenpCNdc80N_DMSO = [ 1 2 3 4 5 9 12 15], if cells 1, 2, 3, 4, 5, 9, 12, and 15 were selected for analysis.
  d. Save the SelectedCells vector as described for the mS structure (6b.) using the name chosen in 6c.
  e. Create a mS structure that contains **only** the cells selected for analysis in step 5a. In the Command window type: mS_Name_sel = mS_Name(SelectedCells_Name), where on the left is the name of the sub-selection.
    ***Note:*** We suggest to name the structure as the mS structure created in 6a **except** ending with _sel: mS_expN_DateOfImaging_ProteinsInChannelOrder_Treatment_sel.**For example:**mS_exp1_13042020_Nnf1CenpC Ndc80N_DMSO_sel = mS_exp1_13042020_Nnf1CenpCNdc80N_DMSO(SelectedCells_exp1_13042020_Nnf1CenpCNdc80N_DMSO). This mS structure now contains only cells 1, 2, 3, 4, 5, 9, 12, and 15.
  f. Save the mS_Name_sel as described for the mS structure (6b.) using the name chosen in 6e.
  g. Spot quality control in one NCS channel – creating a spot selection structure (sS).
    ***Note:*** the spot selection is saved by KiDv1.0.1 as set up in step ii. below at the end of the spot selection. If the function is terminated by the user by simultaneously pressing **Command** and **C** on the keyboard, step g. should be repeated from the beginning.
    i. In the Command window type: sS_Name_sel = kitSelectData(mS_Name_sel, ‘channel’, x, ‘method’, ‘deselect’), where on the left side is the name of the spot selection, and x is the NCS channel under consideration. **For example:** sS_exp1_Nnf1CenpCNdc80N_DMSO_SpotsNdc80N_sel = kitSelectData( mS_exp1_13042020_Nnf1CenpCNdc80N_DMSO_sel, ‘channel’, 3, ‘method’, ‘deselect’)
      ***Note:*** We suggest naming the list as the jobset**except** starting with sS, indicating the channel used for spot selection (SpotsChannel), omitting the date to reduce the length of the name, and ending the structure with _sel: sS_expN_ProteinsInChannelOrder_Treatment_SpotsChannel_sel
      ***Note:*** The ‘deselect’ option allows the user to make a list of all spots that are not suitable for analysis and that will be ignored (bad spots). Use of the ‘select’ option instead of ‘deselect’ allows the user to make a list of the spots suitable for the analysis (good spots).
    ii. KiDv1.0.1 will prompt a window and ask the user to save the sS structure file. Navigate to the folder containing the raw images, input name chosen in i., and press **Save**.
    iii. Next, KiDv1.0.1 will open a figure with all spots that have been paired in the first accepted cell, and their index numbers on top. See Figure 5. Make a table with rows corresponding to each cell analyzed and two columns either for ‘‘bad’’ (ignored) and ‘‘good’’ (selected) spots. Identify ‘‘bad’’ and ‘‘good’’ spots from each cell and write down their index number in the relevant box in the table.

**Figure 5:**
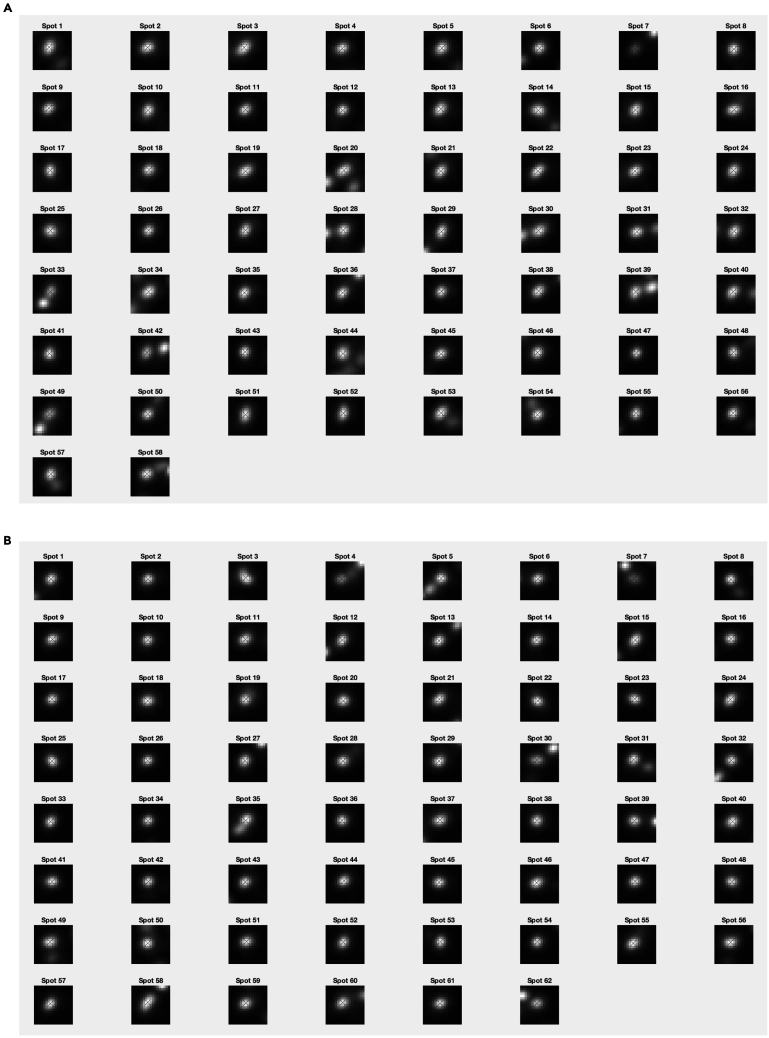
Kinetochore spot selection for spot quality control in ΔEC measurements (A and B) show examples of spot selection GUI using kitSelectData function in two analyzed cells from the exemplar dataset after KiDv1.0.1 spot detection and manual sister-sister pairing. The user is required to evaluate the spot shape and center detection, and to discard non-Gaussian shaped asymmetric spots and spots with poor center detection. A. Spots 1, 31, 40 and 47 are to be discarded. Spot 1 is distorted. Spots 31, 40 and 47 are too close to a neighboring spot and the center may not be detected appropriately. B. Spots 4, 18, 28, 33 and 49 are to be discarded. These spots are distorted.
      **CRITICAL:** classification of “good” and “bad” spots should take into account: (1) the shape of the spot, i.e. expanded or not round spots should be ignored, and (2) the center detection of the spot, i.e. when the cross marking the spot is not in the center, the spot should be ignored. See Figure 5.
      ***Note:*** If no spots were detected or paired in a cell, KiDv1.0.1 will skip that cell.
    iv. In the Command window, the user will be prompted to input either the ‘‘Spots to be ignored’’ or the ‘‘Spots to be selected’’ depending on which method was chosen in step 6g. i. Input the relevant spots by typing in the Command window: [ index numbers of spots to be ignored/selected ]. If no spots are to be ignored/selected, type []. **For example:** in cell1, the user explores signals in Channel 3(Ndc80(N)-647) and ignores spots 3, 7 and 8 by typing: [3 7 8] and pressing **Enter**. In this case, all Ndc80(N)-647 spots detected and paired in cell1 will be selected as ‘‘good’’ spots **except** spots 3, 7 and 8.
    v. Repeat steps iii. and iv. until KiDv1.0.1 displays in the Command window: “Data selection complete”.
    vi. Update metadata in the list of selected spots (sS_sel structure, created in g.) such that cell numbers correspond to raw image cell numbers. In the Command window type: sS_Name = kitUpdateSpotSelections(sS_Name_sel, mS_Name, SelectedCells_Name), where on the left is the name of the updated structure. **For example:** sS_exp1_Nnf1CenpCNdc80N_DMSO_SpotsNdc80N = (sS_exp1_Nnf1-CenpCNdc80N_DMSO_SpotsNdc80N_sel, mS_exp1_13042020_Nnf1CenpCNdc80N_DMSO, SelectedCells_exp1_13042020_Nnf1CenpCNdc80N_DMSO)
      ***Note:*** We suggest naming the updated structure as in 6 g i. **but** omitting the _sel: sS_expN_ProteinsInChannelOrder_Treatment_SpotsChannel
  h. Save the sS structure as described for the mS structure (6b.) using the name chosen in 6 g vi.
  i. Continue to one of the following steps depending on the required analysis: for analysis of intra-kinetochore distances in two-fluorophore detection experiments proceed to step 10; to pool data from experiments with biological replicates perform step 9, and then proceed to step 10 for Intra-kinetochore Delta measurements of the pooled dataset; for spot quality control in three-fluorophore detection experiments proceed to step 7; to make categories based on the presence/absence of a kinetochore marker in the third fluorescent channel in combination with two fluorophore Delta experiments proceed to step 8; for kinetochore intensity measurements proceed to step 11.
7. Kinetochore spot quality control in the second NCS channel to measure the intra-kinetochore distances between three-fluorescently labeled kinetochore markers in pairwise manner. This section illustrates the steps to discard ‘‘bad’’ spots acquired in the second NCS channel when three kinetochore markers are stained. **For example:** the staining is Nnf1-A488, CenpC-A561, Ndc80(N)-A647 and the goal of the experiment is to measure pairwise intra-kinetochore distances between the three markers. Channel2 (CenpC-A561) is the CS channel used for primary spot detection. The NCS channels for spot quality control are Channel1 (Nnf1-A488) and Channel3 (Ndc80(N)-A647). Spot quality control in only one NCS channel, i.e., Channel 3 (Ndc80(N)-647), has been described at step 6. Channel1 (Nnf1-A488) is the second NCS channel to be explored for spot quality here.
  ***Note:*** If the user has closed and re-opened MATLAB, the user needs to load the mS structure saved in step 6b. See step 6b. for instructions.
  a. Inspect spots in the second NCS channel and make a list of the “good”/”bad” spots, also taking into account the NCS channel previously analyzed (step 6).
    ***Note:*** the spot selection is saved by KiDv1.0.1 as set up in step ii. below at the end of the spot selection. If the function is terminated by the user by simultaneously pressing **Command** and **C** on the keyboard, step a. should be repeated from the beginning.
    i. In the Command window type: sS_Name_sel = kitSelectData(mS_Name_sel, ‘channel’, x, ‘method’, ‘deselect’), where, as in step 6 g i., on the left is the name of the spot selection and x is the NCS channel under consideration – choose the NCS channel different than the one analyzed in step 6.
      ***Note:*** We suggest to name the structure as in 6 i. **but** also indicating the second NCS channel in which spot selection will be done here: sS_expN_ProteinsInChannelOrder_Treatment_SpotsChannelSpotsChannel_sel
    ii. KiDv1.0.1 will prompt a window and ask the user to save the sS structure file. Navigate to the folder containing the raw images, input the name chosen in i., and press **Save**.
    iii. KiDv1.0.1 will open a figure with all spots that have been paired in the first accepted cell, and their index numbers on top. Identify the kinetochores to discard in the second **NCS** channel and mark the respective indexes in the table made in step 6 g. iii. (see Figure 5)
      ***Note:*** If no spots were detected or paired in a cell, KiDv1.0.1 will skip that cell.
    iv. In the Command window, the user will be asked to input the “Spots to be ignored”.
      In the Command window type: [indexes of spots to be discarded], where in brackets are the “bad” spots indexes in the first analyzed **NCS** channel (step 6) and the additional “bad” spots identified here. If no spots are to be discarded type [].
      **For example:** in cell 1, spots 3, 7 and 8 were initially ignored because of their signal in Channel 3(Ndc80(N)-647). However, when exploring the signal in Channel1 (Nnf1-A488), spots 3, 4 and 5 were classified as bad spots. The new list of ignored spots will be [3 7 8 4 5] and will result from the union of spots ignored in both NCS channels.
    v. Repeat steps iii. and iv. until KiDv1.0.1 displays in the Command window: “Data selection complete.”
  b. Update metadata in list of selected spots such that cell numbers correspond to raw image cell numbers. In the Command window type: sS_Name = kitUpdateSpotSelections(sS_Name_sel, mS_Name, SelectedCells_Name), where on the left is the name of the updated spot selection. **For example:** sS_exp1_Nnf1CenpCNdc80N_DMSO_SpotsNdc80NNnf1 = kitUpdateSpotSelections(sS_exp1_Nnf1CenpCNdc80N_DMSO_SpotsNdc80NNnf1_sel, mS_exp1_15042020_Nnf1CenpCNdc80N_DMSO, SelectedCells_exp1_15042020_Nnf1CenpCNdc80N_DMSO)
    ***Note:*** we suggest to name the updated spot selection as the one created in step a. **but** omitting _sel: sS_expN_ProteinsInChannelOrder_Treatment_SpotsChannelSpotsChannel
    ***Note:*** The created sS structure specifies the list of spots that have good labeling in both NCS channels and will be used for analysis.
  c. Save the sS structure as described for the mS structure (see step 6b.) but with the name chosen in step 7 b.
  d. Continue to one of the following steps depending on the required analysis: for analysis of intra-kinetochore distances in three-fluorescent channels experiments proceed to step 10; to pool data from experiments with biological replicates perform step 9, and then proceed to step 10 for Intra-kinetochore Delta measurements of the pooled dataset; for kinetochore intensity measurements proceed to step 11.

8. Kinetochore spot categorization. This part illustrates the steps to make categories based on the presence/absence of a kinetochore marker. Kinetochores are categorized as ‘‘positive’’ in the presence of the marker and ‘‘negative’’ when the marker is absent. The analysis is made by eye. These categories can then be used in step 10 to measure Delta distance between the non categorized fluorescent channels at kinetochore marker positive and negative kinetochores. **For example:** the staining is Venus-Mad2, CenpC A561, Ndc80(N)-A647 and the goal is to compare the intra-kinetochore CenpC-A561 to Ndc80(N)-A647 distance at Mad2 positive and negative kinetochores. Channel2 (CenpC-A561) is the CS channel and its spot quality control was done in step 5. Channel3 (Ndc80(N)-A647) is NCS channel and its spot quality control was done in step 6. Channel1 (Venus-Mad2) is the kinetochore marker to be used to categorize positive/negative kinetochores as follows.
  ***Note:*** If the user has closed and re-opened MATLAB, the user needs to load the mS structure saved in step 6b. See step 6b. for instructions.
  a. Making “Marker Positive kinetochores” category.
    ***Note:*** the spot selection is saved by KiDv1.0.1 as set up in step ii. below at the end of the spot selection. If the function is terminated by the user by simultaneously pressing **Command** and **C** on the keyboard, step a. should be repeated from the beginning.
    i. In the Command window type: sS_Name_sel = kitSelectData(mS_Name_sel, ‘channel’, x, ‘method’, ‘select’), where on the left is the name of the spot selection, and x is the channel that will be used to categorize kinetochores. **For example:** sS_exp1_Mad2CenpCNdc80N_DMSO_SpotsNdc80NMad2P_sel = kitSelectData(mS_exp1_2004202_Mad2CenpCNdc80N_DMSO_sel, ‘channel’, 1, ‘method’, ‘select’). KiDv1.0.1 will prompt a window and ask the user to save the sS structure file. Navigate to the folder containing the raw images, input name chosen in i., and press **Save**. Next, KiDv1.0.1 will open a figure with all spots that have been paired in the first accepted cell, and their index numbers on top.
      ***Note:*** We suggest to name the spot selection as the one made in step 6 but indicating the category made: sS_expN_ProteinsInChannelOrder_Treatment_SpotsChannelMarkerP_sel, where “P” stands for “positive”.
    ii. Identify the spots that can be categorized as “positive” and mark their indexes in the column of “good” (selected) spots in the table made at steps 6g. iii.
      ***Note:*** If no spots were detected or paired in a cell, KiDv1.0.1 will skip that cell.
    iii. In the Command window, the user will be asked to input the “Spots to be selected”.
    iv. In the Command window, type: [indexes of spots to be selected], where the indexes of spots to be selected must include only those ones categorized as ‘‘positive’’ **within** the good (selected) spots at step 6g. iii. If no spots are to be selected, type []. **For example:** in cell1, all spots detected and paired in Channel3 (Ndc80(N)-A647) were selected as good spots **except** spots 3, 7 and 8. When exploring Channel1 (Venus-Mad2) signal, spots 1, 2, 6, 8 were categorized as ‘‘positive’’ but spots 8 was in the list of ignored spots. Thus, the list of positive spots will be [1 2 6].
    v. Repeat steps iii. and iv. until KiDv1.0.1 displays in the Command window: “Data selection complete.”
  b. Making “Marker Negative kinetochores” category.
    ***Note:*** the spot selection is saved by KiDv1.0.1 as set up in step ii. below at the end of the spot selection. If the function is terminated by the user by simultaneously pressing **Command** and **C** on the keyboard, step b. should be repeated from the beginning.
    i. In the Command window type: sS_Name_sel = kitSelectData(mS_Name_sel, ‘channel’, x, ‘method’, ‘select’), where on the left is the name of the spot selection, and x is the channel that will be used to categorize kinetochores as negative. **For example:** sS_exp1_Mad2CenpCNdc80N_DMSO_SpotсNdc80NMad2Negative_sel = kitSelectData(mS_exp1_2004202_Mad2CenpCNdc80N_DMSO_sel, ‘channel’, 1, ‘method’, ‘select’)
      ***Note:*** We suggest to name the spot selection as the one made in step 6g i. but indicating the category made: sS_expN_ProteinsInChannelOrder_Treatment_SpotsChannelMarkerN_sel, where “N” stands for “negative”.
    ii. KiDv1.0.1 will prompt a window and ask the user to save the sS structure file. Navigate to the folder containing the raw images, input name chosen in i., and press **Save**.
    iii. Next, KiDv1.0.1 will open a figure with all spots that have been paired in the first accepted cell, and their index numbers on top. Identify the spots that can be categorized as “negative” and mark their indexes in the column of “good” (selected) spots in the table made at step 6g. iii.
      ***Note:*** If no spots were detected or paired in a cell, KiDv1.0.1 will skip that cell. Identify the spots that can be categorized as “negative”.
    iv. In the command window, the user will be asked to input the ‘‘Spots to be selected’’. In the Command window type: [indexes of spots to be selected], where the indexes of spots to be selected must include only those ones categorized as ‘‘negative’’ **within** the good (selected) spots at step 6 g. iii. If no spots are to be selected type []. **For example:** in cell1, all spots detected and paired in Channel3 (Ndc80(N)-A647) were selected as good spots **except** spots 3, 7 and 8. When exploring Channel1 (VenusMad2) signal, spots 3, 4, 5, 9, 10 were categorized as ‘‘negative’’ but spots 3 was in the list of ignored spots. Thus, the list of negative spots will be [4 5 9 10].
    v. Repeat steps iii. and iv. until KiDv1.0.1 displays in the command window: “Data selection complete.”
  c. Update metadata in list of selected spots such that cell numbers correspond to raw image cell numbers. Here, the user will update both the list of marker “positive” and of marker “negative” kinetochores.
    i. Updating the list of Marker ‘‘positive’’ kinetochores. In the Command window type: sS_Name= kitUpdateSpotSelections(sS_Name_sel, mS_Name, SelectedCells_Name), where on the left is the name of the updated spot selection. **For example:** sS_exp1_Mad2CenpCNdc80N_DMSO_SpotNdc80NMad2P = kitUpdateSpotSelections (sS_exp1_Mad2CenpCNdc80N_DMSO_SpotsNdc80NMad2P_sel, mS_exp1_2004202_Mad2CenpCNdc80N_DMSO, SelectedCells_exp1_2004202_Mad2 CenpCNdc80N_DMSO)
      ***Note:*** we suggest to name the update spot selection as the one created in step a. **but** omitting _sel: sS_expN_ProteinsInChannelOrder_Treatment_SpotsChannelMarkerP
    ii. Updating the list of Marker ‘‘negative’’ kinetochores. In the Command window type: sS_Name = kitUpdateSpotSelections(sS_Name_sel, mS_Name, SelectedCells_Name), where on the left is the name of the updated spot selection. **For example:** sS_exp1_Mad2CenpCNdc80N_DMSO_SpotsNdc80NMad2N = kitUpdateSpotSelections (sS_exp1_Mad2CenpCNdc80N_DMSO_SpotsNdc80NMad2N_sel, mS_exp1_2004202_Mad2CenpCNdc80N_DMSO, SelectedCells_exp1_2004202_Mad2CenpCNdc80N_DMSO). Save the sS structures as described for the mS structure (see step 6b.) but with the name chosen in 8 c i. and ii.
      ***Note:*** we suggest to name the update spot selection as the one created in step b. **but** omitting _sel: sS_expN_ProteinsInChannelOrder_Treatment_SpotsChannelMarkerN
      ***Note:*** The following two lists of spot selection categories were created in this step: (1) Marker positive. Here, the user created a list of spots that are positive for the kinetochore marker of interest, and have good spot quality in the NCS channel that will be used for Delta measurements. That list has the suggested name of: sS_expN_ProteinsInChannelOrder_Treatment_SpotsChannelMarkerP; and (2) Marker negative. The user also created a list of spots that are negative for the kinetochore marker of interest and have good spot quality in the NCS channel that will be used for Delta measurements. That list has the suggested name of: sS_expN_ProteinsInChannelOrder_Treatment_SpotsChannelMarkerN
  d. Continue to one of the following steps depending on the desired analysis: for analysis of intra-kinetochore Delta between two fluorescently labeled kinetochoremarkers at kinetochores ‘‘positive’’ and ‘‘negative’’ for a third fluorescently labeled kinetochore marker proceed to step 10; to pool data from experiments with biological replicates perform step 9 and then proceed to step 10 for Intra-kinetochore Delta measurements; for intensity measurements proceed to step 11.

### Pooling the data of experiment biological replicates for kinetochore intra-measurements

**Timing: 1–2 h**

The BEDCA correction algorithm requires a minimum of 300 kinetochores to reliably correct the inflation (based on our experience with our fluorophores), depending on the (true) distance and other factors (see BEDCA, step 12). To increase the number of kinetochores for an experimental set up, several biological replicates can be pooled together.

**CRITICAL:** The experiments to be pooled must be biological replicates.

9. Pooling of the data. The kinetochore intra-measurement function (dublIntraMeasurements) requires as input the mS structure (movie structure, created in step 6) and the sS structure (spot selection, step 6, 7 or 8), see step 10. Therefore, to make a unique intra-kinetochore measurement output for several experiment repeats, the mS and sS structures of the individual experiments (each created by following the steps above) are pooled. The pooled mS and sS structures are analyzed with the dublIntraMeasurments function (step 10).
  a. Pooling of the sS structures. Pool the sS structures by typing the following command: sS_Pool_Name = kitCombineSpotSelections({sS_Name, sS_Name, sS_Name}), where on the left is the name of the pooled structure and in curly brackets are the sS structures to be pooled from different experimental repeats.
    **CRITICAL:** If the sS structures to be pooled do not appear in the user Workspace window, load the sS structures, one by one, by dragging the file from the folder with raw data into the Workspace window. The sS structure will be loaded with name spotSelection. Rename the variable by typing in the Command Window: sS_Name = spotSelection, where sS_Name is the name under which the sS structure was saved.
    ***Note:*** We suggest the pooled structure to be named as sS_Pool_expN_expN_expN_ProteinsInChannelOrder_Treatment_SpotsChannels, i.e. as the individual sS structures but indicating which experiments have been pooled.
  b. Pooling of the mS structures. In the command window type: mS_Pool_Name = {mS_Name, mS_Name, mS_Name}, where on the left is the name of the pooled mS structure, and in the curly brackets are the mS structures to be pooled from different experiment repeats.
    **CRITICAL:** If the mS structures to be pooled do not appear in the user Workspace window, load the mS structures by dragging the file from the folder with raw data into the Workspace window.
    ***Note:*** {} is termed curly brackets.
    **CRITICAL:** the order of sS and mS structures in a. and b. with respect to which experiment is analyzed **must** be the same.
    ***Note:*** We suggest to name the mS structure of the pooled experiments as mS_Pool_expN_expN_expN_ProteinsInChannelOrder_Treatment, i.e. as the individual mS structures but omitting the date and indicating which experiments have been pooled.
  c. Save the structure(s) as described in step 6b with the appropriate names.
  d. To make intra-kinetochore measurements of the pooled data (iM), in step 10 use as input the created pooled mS and sS structures and the options that were used for analysis of the individual experimental repeats.

### Intra-kinetochore measurements, basic raw Delta statistics, and plots

**Timing: 1–3 h**

10. Making Intra-kinetochore measurements. Delta is measured between two channels. For three-channel datasets, Delta is measured between each pair of channels. For marker positive/negative datasets (Delta of a category of spots), Delta is measured between the non-categorized fluorescent marker channels at marker “positive” and “negative” kinetochores.
  ***Note:*** If the mS and sS structures that the user has created before do not appear in the workspace, the user needs to load the structures as described in steps 6b. and 6d.
  ***Note:*** The following steps can be performed for an individual experiment (continuing from step 8.) or a pool of experiment repeats (after performing step 9.).
  a. Measure Delta. In the Command window type: iM_Name = dublIntraMeasurements(mS_Name, ‘centralise’, 1, ‘channels’, [a b], ‘spotSelection’, sS_Name), where on the left is the name of the intra-kinetochore measurements variable, a and b are the channels between which Delta will be measured, and sS_Name is the list of selected spots created in step 6, 7 or 8.
    **CRITICAL:** In the name include the treatment name flanked by underscores. If no treatment is applied use: ”_untreated_”. Annotation of treatment is critical for data recognition by the BEDCA software.
    ***Note:*** We suggest to name the intra-kinetochore measurement structure as follows: iM_expN_ProteinsInChannelOrder_Treatment_ProteinProtein, where ProteinProtein are the two proteins between which Delta is measured. **For example:**iM_exp1_Nnf1CenpCNdc80N_DMSO_CenpCNdc80N
    ***Note:*** Add _MarkerP or _MarkerN if you are analyzing a dataset of marker positive/negative kinetochores (continuing here from step 8). **For example:**iM_exp1_Mad2-CenpCNdc80N_DMSO_CenpCNdc80N_Mad2P for Mad2 positive kinetochore dataset.
  b. Explanation of the options:
    i. ‘centralise’, 1 will prompt KiDv1.0.1 to use data centralization. Centralization is the fine chromatic shift correction. Here, KiDv1.0.1 detects the center of mass of the analyzed kinetochores in each channel, and aligns the centers of mass in the different channels to correct the chromatic shift. Always switch on the centralization for this method.
    ii. ‘channels’, [a b] This option defines the channels between which the user will measure Delta. If the user is measuring Delta between channels 2 and 3, the user needs to specify: ‘channels’, [2 3]
    iii. ‘spotSelection’, sS_Name This option allows the user to specify a list of kinetochores for which the measurement is to be done. For vector use the sS list made in step 6 for two-channel Delta measurement, in step 7 for three-channel Delta measurement, and in step 8 for Delta measurement of a spot category.
  c. **Examples**:
    i. Two channel Delta measurement – use the sS list from step 6: **Type:** iM_exp1_ Nnf1CenpCNdc80N_DMSO_CenpCNdc80N = dublIntraMeasurements(mS_exp1_13042020_ Nnf1CenpCNdc80N_DMSO, ‘centralise’, 1, ‘channels’, [2 3], ‘spotSelection’, sS_exp1_Nnf1CenpCNdc80N_DMSO_SpotsNdc80NNnf1)
    ii. Three channel Delta measurement – use the sS list from step 7 and do step (1) for the three combinations: **Type:** iM_exp1_Nnf1CenpCNdc80N_DMSO_Nnf1CenpC = dublIntraMeasurements(mS_exp1_13042020_Nnf1CenpCNdc80N_DMSO, ‘centralise’, 1, ‘channels’, [1 2], ‘spotSelection’, sS_exp1_Nnf1CenpCNdc80N_DMSO_SpotsNdc80NNnf1) **Type: iM_exp1_Nnf1CenpCNdc80N _DMSO_CenpCNdc80N = dublIntraMeasurements(mS_exp1_13042020_Nnf1CenpCNdc80N_DMSO, ‘centralise’, 1, ‘channels’, [2 3], ‘spotSelection’, sS_exp1_Nnf1CenpCNdc80N_DMSO_SpotsNdc80NNnf1) Type: iM_exp1_Nnf1CenpCNdc80N_DMSO_Nnf1Ndc80N = dublIntraMeasurements(mS_exp1_13042020_Nnf1CenpCNdc80N_DMSO, ‘centralise’, 1, ‘channels’, [1 3], ‘spotSelection’, sS_exp1_Nnf1CenpCNdc80N_DMSO_SpotsNdc80NNnf1)**
    iii. Delta measurement for Spot category – use the sS lists from step 8 and do step (1) for each category: **Type:** iM_exp1_Mad2CenpCNdc80N_DMSO_CenpCNdc80N_Mad2P = dublIntraMeasurements(mS_exp1_2004202_Mad2CenpCNdc80N_DMSO, ‘centralise’, 1, ‘channels’, [1 2], ‘spotSelection’, sS_exp1_Mad2CenpCNdc80N_DMSO_SpotsNdc80NMad2P) **Type:** iM_exp1_Mad2CenpCNdc80N_DMSO_CenpCNdc80N_Mad2N = dublIntraMeasurements(mS_exp1_2004202_Mad2CenpCNdc80N_DMSO, ‘centralise’, 1, ‘channels’, [1 2], ‘spotSelection’, sS_exp1_Mad2CenpCNdc80N_DMSO_SpotsNdc80NMad2N)
  b. Quick statistics and plots of the non-inflation corrected Delta measurements and kinetochore pair statistics.
    i. To quickly access the mean, median and standard deviation of these measurements per kinetochore, type: dublBasicStats(iM_Name), where in brackets is the iM name given in step 10a. KiDv1.0.1 will display a list of the characteristics in the Command window. Choose the desired measurement and type its number in the Command window. **For example:** to access the non-corrected Delta 3D statistics, type 1 and press **Enter**.
    ii. To quickly visualize a plot of these measurements per kinetochore, type: dublBasicPlots(iM_Name) and press **Enter**, where in brackets is the iM name given in step 10a. KiDv1.0.1 will display a list of the characteristics in the Command window. Choose the desired measurement and type its number in the Command window. **For example:** to plot the non-corrected Delta 3D, type 1 and press **Enter**.
  c. Accessing the non-inflation corrected Delta and kinetochore pair data saved in the iM structures created in step 10a. The detected kinetochore positions and distances are saved in the iM_Name.microscope structure.
    **CRITICAL:** The inflation of the Delta measurements in the iM structures makes them non-informative *per se* and is to be used only for comparison to other studies that use non-inflation corrected measurements.
    i. To access the kinetochore coordinates use .coords, followed by .x .y or .z for the corresponding dimension. This will render a list with four columns, where the first two columns are the coordinates of the inner and outer markers of one of the sister kinetochores, and the third and fourth column are the inner and outer marker coordinates at the second sister kinetochore. **For example:** to access the x coordinate of Nnf1 (column 2 and 4) and CenpC (column 1 and 3), type: iM_exp1_Nnf1CenpCNdc80N_DMSO_Nnf1CenpC.microscope.coords.x
    ii. To access the Delta 1D measurement vector with Delta>200nm filtered out, type: iM_Name.microscope.depthFilter.delta.oneD
    iii. To access the Delta 2D measurement vector with Delta>200nm filtered out, type: iM_Name.depthFilter.delta.twoD.all
    iv. To access the Delta 3D measurement vector with Delta>200nm filtered out, type: iM_Name.microscope.depthFilter.delta.threeD.all
    v. To access the raw Delta 1D measurement vector, type: iM_Name.raw.microscope.delta.oneD
    vi. To access the raw Delta 2D measurement vector, type: iM_Name.raw.microscope.delta.twoD.all
    vii. To access the raw Delta 3D measurement vector, type: iM_Name.raw.microscope.delta.threeD.all
    viii. To access the 3D kinetochore sister-sister separation, type: iM_Name.microscope.sisSep.threeD

### Intensity measurements

**Timing: 1–3 h**

11. Measurement of kinetochore marker fluorescent intensity. Here, we describe how to perform automated kinetochore intensity measurements in KiDv1.0.1 and the different options that can be used.
  ***Note:*** KiDv1.0.1 has the following intensity measurement functionalities: (1) KiDv1.0.1 measures the intensity in a sphere with 300 nm radius (default, other mask and radius can be specified in the jobset, see step 4 d iv.) centered at the detected kinetochore centers; and (2) KiDv1.0.1 uses the detected kinetochore centers of a reference kinetochore marker in one channel to measure the intensity in a sphere with the same center and 300 nm radius (default, other mask and radius can be specified in the jobset, see step 4 d iv.) in another channel. **For example:** the staining is Ndc80(C)-eGFP, CenpC-A561 and Ndc80(N)-A647. The goal is to measure the intensity of Mad2 at kinetochores. Channel2 (CenpC-A561) is the CS channel used for primary spot detection. Kit can measure the intensity of Mad2 in a sphere with 300nm radius centered at the detected centers of CenpC kinetochores.
  a. In the Command window type: IntiM_Name = dublIntensityMeasurements(mS_Name, ‘channels’, [a b], ‘paired’, 0 or 1, ‘refMarker’, ‘option’, ‘spotSelection’, sS_Name), where on the left is the name of the intensity measurement structure, mS_Name is the mS structure generated in step 6 or step 9, and sS_Name is the spot selection made in step 6, 7, 8, or 9. For explanation of the options see the following sub-steps.
    ***Note:*** We suggest to name the intensity measurement IntiM_expN_ProteinsInChannelOrder_Treatment_Marker1Marker2, where Marker1 and Marker2 are two channels in which the user will measure intensity. **For example:**IntiM_exp1_Nnf1CenpCNdc80N_DMSO_Nnf1CenpC
    i. Options: Channels. In the above function, a and b are the channels in which KiDv1.0.1 will measure the intensity. **For example:** ‘channels’, [1 2] indicates channels 1 and 2.
    ii. Options: Paired. ‘paired’, 1 will prompt kit to use only the kinetochores paired in step 5. ‘paired’, 0 will prompt kit to use all spots detected irrespective of previous pairing.
    iii. Options: RefMarker. ‘refMarker’ – ‘self’ prompts KiDv1.0.1 to measure the intensity of the indicated channels in a mask centered at the detected kinetochore center. The mask is defined in the jobset. The default mask is a sphere with 300 nm radius, see step 4 d iii. ‘inner’ or ‘outer’ prompts KiDv1.0.1 to use the spot centers detected either in the inner or the outer of the two given channels (the user stated the order of kinetochore markers in step 4. f. iii.) to measure the intensity in a mask in both ‘channels’. As above, the mask is defined in the jobset. The default mask is a sphere with 300 nm radius, see step 4 d ii. **For example:** the staining is Nnf1-A488, CenpC-A561 and Ndc80(N)-A647. (1) The goal is to measure the intensity of Nnf1 around kinetochores detected in the CenpC channel. CenpC is positioned inner of Nnf1. Use ‘refMarker’, ‘inner’ to measure the intensity of Nnf1 in a sphere with 300 nm radius, centered at the detected spot centers of CenpC kinetochores. (2) The goal is to measure the intensity of CenpC around kinetochores detected in the Nnf1 channel. Nnf1 is positioned outer of CenpC. Use ‘refMarker’, ‘outer’ to measure the intensity of CenpC in a sphere with 300 nm radius, centered at the detected spot centers of Nnf1 kinetochores. (3) The goal is to measure the intensity of CenpC at kinetochores detected in the CenpC channel. Use ‘refMarker’, ‘self’ to measure the CenpC intensity in a sphere with 300 nm radius, centered at the detected spot centers of CenpC kinetochores. If the user wishes to measure the intensity of a selection of kinetochores made in the ‘‘Spot quality control’’ section (step 6, 7, 8, or 9), the user can state the sS list at the place of sS_Name. Otherwise, use [] (an empty vector) instead of sS_Name.
  b. Accessing the intensity measurements. The IntiM structures, created in step 11a. contains the background and the background subtracted kinetochore intensity measurements in the selected fluorescent channels.
    i. To access the inner kinetochore marker background intensity type: IntiM_Name.intensity.bg.inner **For example:** To access the background intensity of the inner kinetochore marker (CenpC), type: IntiM_exp1_Nnf1CenpCNdc80N_DMSO_Nnf1CenpC.intensity.bg.inner
      ***Note:*** The measured background is global – a mean of all of the pixels in the stack.
    ii. To access the inner kinetochore marker mean background-subtracted intensity measurements (per kinetochore), type: IntiM_Name.intensity.mean.inner
    iii. To access the inner kinetochore marker maximum background-subtracted intensity measurements (per kinetochore), type: IntiM_Name.intensity.max.inner
    iv. To access the outer kinetochore marker background intensity measurements (per kinetochore), type: IntiM_Name.intensity.bg.outer
    v. To access the outer kinetochore marker mean background-subtracted intensity measurements (per kinetochore), type: IntiM_Name.intensity.mean.outer
    vi. To access the outer kinetochore marker maximum background-subtracted intensity measurements (per kinetochore), type: IntiM_Name.intensity.max.outer

### Bayesian Euclidian distance correction algorithm (BEDCA)

**Timing: 2–3 h for step 13**

**Timing: 2–5 h for step 14**

**Timing: 2–4 h for step 15**

Distances between fluorescent spots are inflated (Churchman et al., 2006), and thus need correction. In 3D and with an anisotropic PSF there is no analytical method. BEDCA is a sampling algorithm providing samples of the inferred true distance which can be used to generate a posterior distribution. BEDCA is a Markov chain Monte Carlo (MCMC) algorithm and the Bayesian framework in BEDCA gives tighter confidence intervals than previous methods (Churchman et al., 2005, Churchman et al., 2006, Suzuki et al., 2018; see Figure1, for testing of BEDCA see Roscioli et al., 2020)

**CRITICAL:** In order to produce reliable results, the kinetochore and cell sample sizes need to be sufficiently large, the latter to ensure biological variability is taken into account. We recommend cell sample sizes greater than 5 (ideally as quantified by the diversity measures to allow for unequal kinetochore counts). The kinetochore sample size should be a few hundred based on our experience (with our experimental system); provided the posteriors are approximately Gaussian the required sample size N can be estimated from an initial run with N_1_ samples, length variance var_e_, and desired length variance var_d_ as N=N_1_ x var_d_/var_e_. Sample sizes need to be increased for (true) distances in the range of 0–30 nm, for instance a sample size of 500 kinetochores may be required in this range. In this range, posterior length distributions may have a thick tail towards 0; the posterior standard deviation is then not a good estimate of the confidence interval and the above calculation likely underestimates the required sample size. To make sure the correction parameters and sample size are appropriate, the user should always check the quality of the correction by following the advice in step 14.

**Figure 6:**
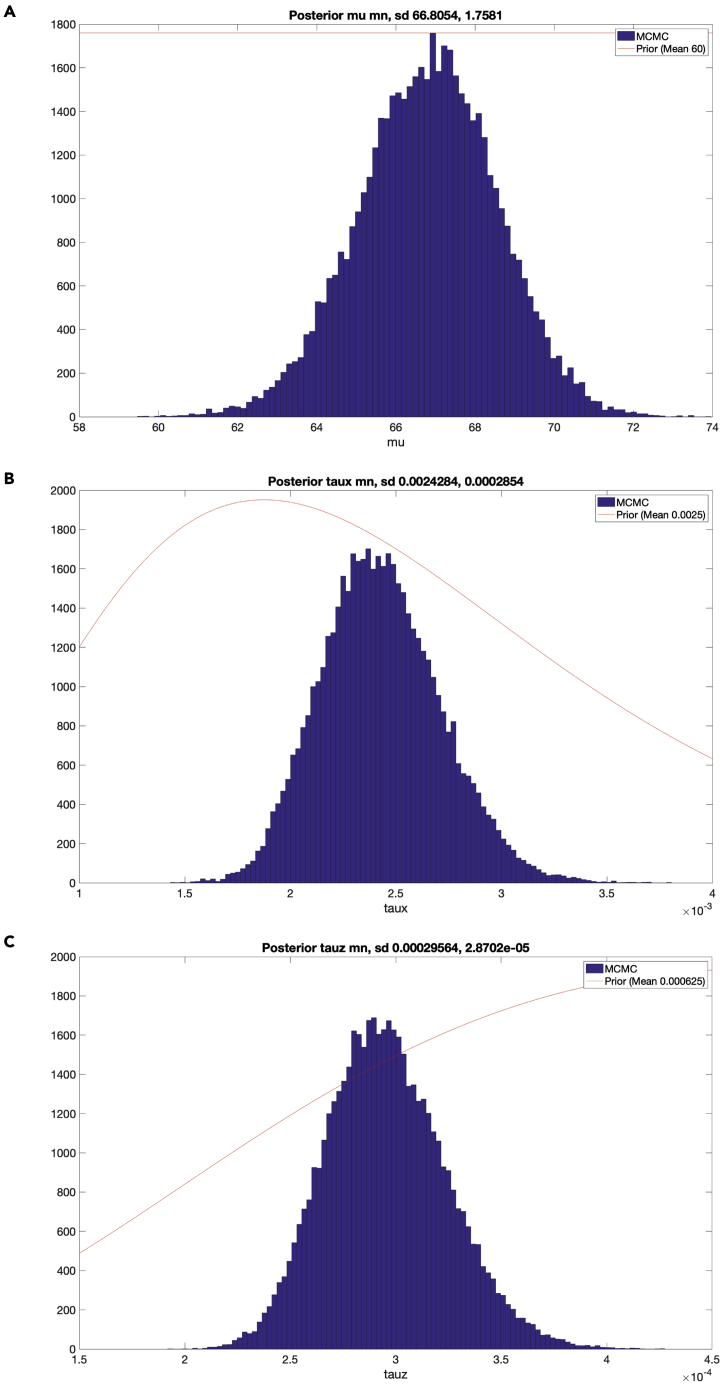
Quality control of BEDCA run Posterior distributions of the ΔEC mean, taux, and tauz (blue) and the corresponding prior distributions (red). (A) Example of figure saved as “Posterior_mu” in “MCMC_EuclDistMargFigs” folder using Nnf1-to-Ndc80N distance correction from provided dataset. Histogram of corrected mean (mu) distance (blue). Red line is the corresponding prior. Prior mean is indicated in the legend (60 nm). The mean and sd. of the posterior mu distribution are stated above (mean 57.4496 nm, sd. 1.214 nm). (B) Example of figure saved as "Posterior_taux” in “MCMC_EuclDistMargFigs” folder, dataset as in A. Histogram of posterior precision in x(y) (taux) (blue). Red line is the corresponding taux prior. Prior mean is stated in the legend (0.0025 nm^-2^). The mean and sd. of the posterior taux distribution are stated above (mean 0.002594 nm^-2^, sd. 0.00021498 nm^-2^). (C) Example of figure saved as "Posterior_tauz” in “MCMC_EuclDistMargFigs” folder, dataset as in A. Histogram of precision in z (tauz) (blue). Red line is the corresponding tauz prior. Prior mean is stated in the legend (0.000625 nm^-2^). The mean and sd. of the posterior tauz distribution are stated above (mean 0.00036346 nm^-2^, sd. 0.000023406 nm^-2^).
    iv. Chains. By default, BEDCA uses four independent runs (chains), i.e., running on the same data input. This allows convergence of the algorithm to be assessed. Sampling algorithms need to converge to the desired distribution. In absence of convergence the results are **UNRELIABLE.** Convergence is thus essential for accurate distance correction.
    v. Number of steps. The number of steps is the number of iterations BEDCA uses of the underlying Markov chain. In case of a lack of convergence, the number of steps should be increased and the algorithm re-run. It is advised to increase by multiples of 2 or 5. Convergence is slower for smaller data sets and smaller distances.
  b. Filters
    i. Z filter. The z filter is optional and removes any distances that exceed 100 nanometers projected distance in the z-plane. This filtered data is only used for comparison plots such that the user can check for effects on the distribution from kinetochores lying in the z-plane where the resolution is poorer as compared to the x/y plane.
    ii. 3D distance filter. Any uncorrected 3D Delta distances above 200 nm are filtered out by default. Delta distances rarely go beyond 200 nm given the size of the kinetochore (although corona proteins may be this distance from the CCAN), and thus such measurements are outliers in the data. The 3D Delta filter by default removes KT measurements above this threshold (see step 14 b.). The user can increase the distance filter to an appropriate value by replacing 200 with a desired distance in nanometers.

**Figure 7:**
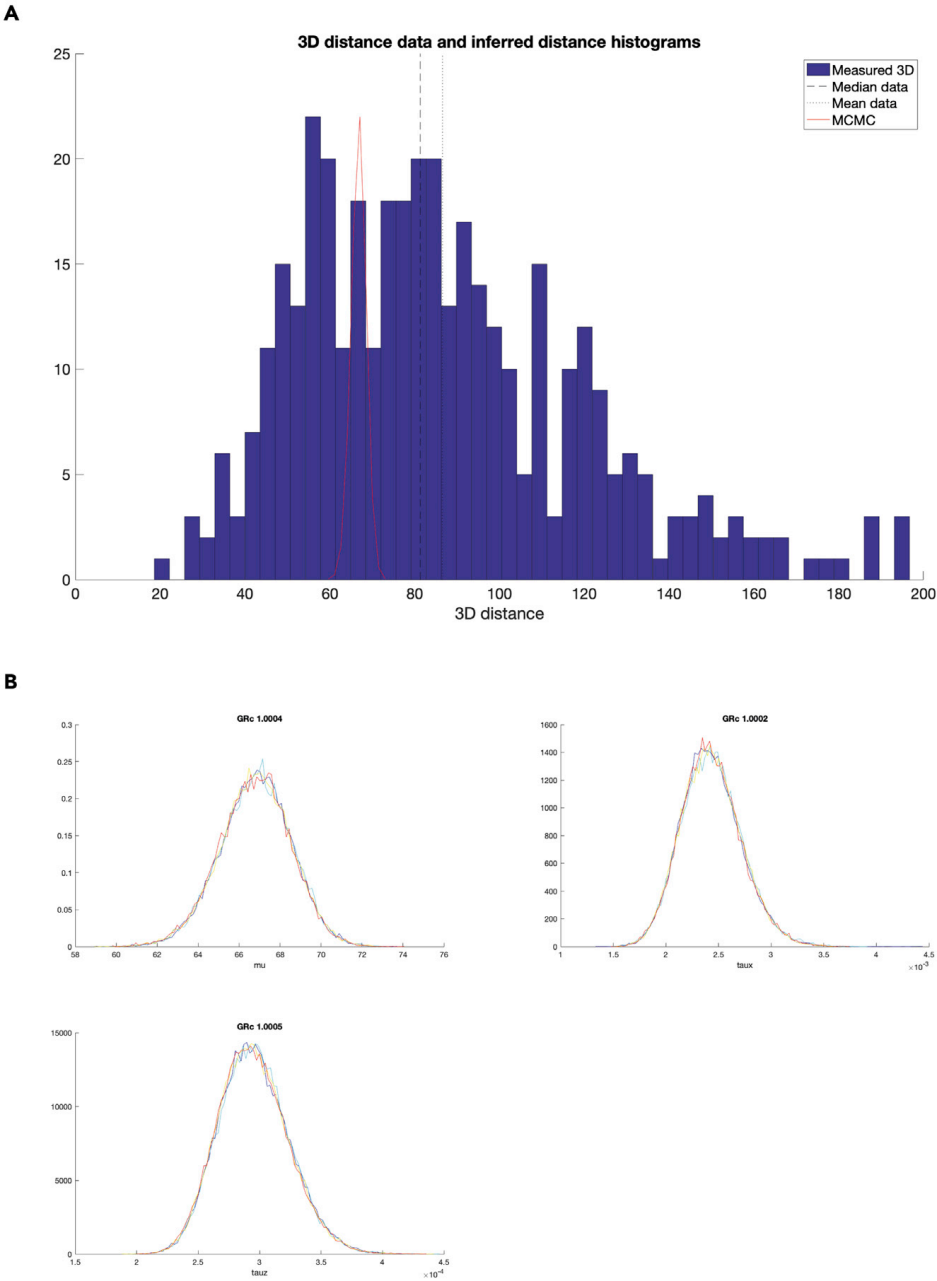
Quality control of BEDCA run Δ3D and ΔEC distributions, and convergence. (A) 3D Delta Euclidian distance distribution of the Nnf1-to-Ndc80N distance from the provided dataset. The figure is automatically saved by the algorithm under the name “DataWithPostEsts”. The figure shows histogram of the 3D distance measurements before (nm, blue) and after (nm, red) BEDCA correction.The uncorrected 3D Delta distribution (blue) lies to the left of the 200 nm (default) cutoff imposed in BEDCA. (B) Convergence of the mean, taux (precision in x), and tauz (precision in z) of the Nnf1-to-Ndc80N distance measurements from the provided dataset (see A.). The figure is automatically saved as “ConvPosteriorSummary”. BEDCA is ran with default parameters. GRc numbers below 1.01 indicate successful convergence. Taux has order of magnitude 10^-3^ nm^-2^. Tauz has order of magnitude 10^-4^ nm^-2^.
    ii. Evaluate the convergence. See Figure 7B. Figure with the convergence characteristics is automatically saved in MATLAB\MCMCruns\name\Figures under the name ‘‘ConvPosteriorSummary’’. Here, name refers to the name under which the analysis is saved (see step 12 b. v.). The Figure displays three plots: the convergence of the mean (mu), of taux (taux) and tauz (tauz) (for more information on priors and convergence see steps 13 a). First, check the convergence value is displayed at the top of each plot. If the convergence value (GRc) for all parameters is below 1.02, convergence has been successful and the user can proceed to step (3).Otherwise, the algorithm has not converged and the results are **UNRELIABLE**. If not converged, set the Number of steps (see step 13 a. v.) to a higher value, i.e., if the user has ran BEDCA with ‘nsteps’, 100000, the user could attempt 300000. The maximum number of iterations that we have used for problematic datasets is 1,000,000. If that does not lead to convergence, increase the sample size if it is below 1000 kinetochores as too small kinetochore and cell sample size can also lead to lack of convergence. Next, check if the estimated taux is smaller than tauz. Taux and tauz quantify the detection accuracy in the x/y and z axes, respectively. The resolution in x/y is in principle better than the one in z. Thus, the error in the x/y direction (taux) should be smaller than the error in the z-direction (tauz). In Roscioli et al., we observed orders of taux to be 10^-3^ nm^2^ and for tauz 10^-4^ nm^-2^. Finally, check the shape of the mean, taux and tauz distributions. The distributions should not display multiple peaks or have a shape strongly deviating from Gaussian.
      ***Note:*** If the true Euclidian distance is very close to 0, the Gaussian distribution of the mean Euclidian distance (mu) will be centered close to 0 value and thus the user will see only the positive values of the distribution, i.e. the distribution should resemble Gaussian distribution cut-off at 0 on the left side. Such appearance is normal and the user should not classify this scenario as abnormal distribution shape. However, (true) small distances (of the order of the spot centre errors) can have thick tails running towards zero. Higher kinetochore sample sizes are recommended.
  b. Accessing the data
    i. Accessing the corrected distribution of Delta values. The distribution after Euclidian correction is saved as .csv file under the name of “MCMCSamples_chain1” in MATLAB\MCMCruns\AnalysisName, where AnalysisName refers to the name under which the analysis is saved (see step 12 b. v.).
    ii. Accessing the statistics of the corrected Delta distribution and the statistics of the BEDCA run. The statistics of the distribution mean, taux and tauz (see step 13 a iii.), and estimated sdx and sdz is saved as .csv file under the name of “SummaryStats_chain1” in MATLAB\MCMCruns\AnalysisName (see Figure 8), where AnalysisName refers to the name under which the analysis is saved (see step 12 b. v.).

12. Input the 3D delta information from step 10. into the BEDCA algorithm
  a. Input the iM file in the BEDCA directory: Load the iM structures saved in step 10 into MATLAB by dragging them in the Workspace window.
    i. Analysis of the same Delta distance in samples with more than one treatment done in parallel. If more than one treatment is analyzed, in the Workspace window select all treatments that correspond to the same experiment and the same protein-protein distance by holding down **Command** and clicking on each iM structure with the left mouse button. Then save the structure selection in one file by clicking with right mouse button on the selection and choosing **Save as**. Choose as destination MATLAB/BEDCA/ExptData and as name choose the name of the individual iM structures **without** Treatment annotation (the structures saved together should be of the same distance, same experiment but different treatment only, see example below). **For example:** the Ndc80N-CenpC distance is measured in cells treated with DMSO and with nocodazole, creating the iM structures: iM_exp1_Nnf1CenpCNdc80N_DMSO_CenpCNdc80N and iM_exp1_Nnf1CenpCNdc80N_noc_CenpCNdc80N. As all other parameters are the same except for treatment, the two structures can be saved in a single file, named iM_exp1_Nnf1CenpCNdc80N_CenpCNdc80N. Then the single file is input into BEDCA as described above, and each treatment is accessed in BEDCA using the ‘‘treatment’’ input in the Run_BEDCA function.
    ii. Analysis of experiment with only one treatment. If only one treatment is used for the analyzed distance, in the Workspace window click with right mouse button on the iM structure, click on **Save as**, for name type the iM structure name **without** Treatment annotation (see example below) and for saving directory choose: MATLAB/BEDCA/ExptData. Repeat that for all iM structures to be corrected, i.e., all protein-protein distance measurements. **For example:** The iM structure created in step 10 is named iM_exp1_Nnf1-CenpCNdc80N_DMSO_CenpCNdc80N. Save the structure as: iM_exp1_Nnf1CenpCNdc80N_DMSO_CenpCNdc80N. The structure will still appear in the Workspace window with name iM_exp1_Nnf1CenpCNdc80N_DMSO_CenpCNdc80N while the file will be named iM_exp1_Nnf1CenpCNdc80N_ CenpCNdc80N.
      **CRITICAL:** Do not include annotation of the treatment in the iM structure saved in the ExptData folder. In case of multiple treatments, the iM structure contains all treatments of the **same** intra-kinetochore pair measurement in an individual or pooled experiment.
      ***Note:*** We suggest the user to name this structure file iM_expN_ProteinsInChannelOrder_ProteinProtein, denoting the experiment number, the proteins imaged in channel order and the protein-protein distance.
  b. Input information about the data location and characteristics to BEDCA.
    i. Open the BEDCA folder in the MATLAB Current Folder window by double-clicking with the right mouse button.
    ii. Open the ExptData_list file in the same manner. The ExptData_list file will appear in the Editor window. Here, the user enters information about the location of the data and the staining.
    iii. Add any treatments used for first time with the software in the treatments library: At the top of the list the user will see TreatmentLib = {{‘DMSO’, ‘DMSO’}, {‘DMSO’, ‘untreated’}, {'Taxol15min','tax15min','tax'} …}. To add a new treatment, after the first curly bracket, type in curly brackets the ‘Treatment’ and any synonyms used, separated by commas (see example in the file and in the previous sentence). Make sure the treatment name input matches exactly the treatment name in the iM structure, saved in step 10. The treatments that have already been included can be seen in the curly brackets after TreatmentLib.
    iv. Indicate the iM file name saved in the ExptData folder that will be used for correction: FileNames{n} = ‘iM_expN_ProteinsInChannelOrder_ProteinProtein.mat’;, where n is the item number in the list and the expression after is the name of the iM file.
      ***Note:*** The user can leave comments in the list by using percentage sign before the text. That text will appear in green and will not be read as code by MATLAB.
    v. Indicate the name of the analysis. To do so navigate to the ExptDats section. Provide the name of the sample by typing: Expt_Dats{n}.name = AnalysisName, where n is the item number in the list as chosen in 12 b. iv. and the expression after is the name of the Delta distances analysis. After running, BEDCA will automatically create a folder named as the AnalysisName input that will contain all figures and data of the BEDCA run. The analysis folder will be situated within the “MCMCruns” folder that is automatically created in the “MATLAB” folder after the first BEDCA run.
    vi. Indicate the treatment. To do so navigate to the Treatments section of the ExptData_list file. At the bottom of the section type: ExptDats{n}.treatment = ‘Treatment’;, where n is the item number in the list as chosen in step 12 b. iv. , and ‘Treatment’ is the treatment of the sample as saved in the iM structure(s) (created in step 10.) and in the TreatmentLib (step 12 b. iii.). If the input iM file (created in step 12 a.) contains more than one treatment (see step 12. a.), type ExptDats{n}.treatment = ‘Treatment’; for each treatment, keeping the same n number as chosen in step 12 b. iv. **For example:** In step 10, the user created the structures: iM_exp1_Nnf1CenpCNdc80N_ DMSO_Nnf1CenpC and iM_exp1_Nnf1CenpCNdc80N_tax_Nnf1CenpC. In step 12 a., the user created a combined iM file named: iM_exp1_Nnf1CenpCNdc80N_Nnf1CenpC. For this example, the item number of the distance is 1. To indicate the treatments, the user is required to type: ExptDats{1}.treatment = ‘DMSO’; ExptDats{1}.treatment = ‘tax’;
    vii. Indicate the source of fluorescence in the sample. Navigate to the Staining section of the ExptData_list file and type: ExptDats{n}.fluorophore = ‘stain’;, where n is the item number in the list as chosen in step 12 b. iv. , and ‘stain’ is ‘Ab’ if both proteins are labeled via indirect immunofluorescence (antibody staining) and ‘GFP’ if one or both of the proteins are labeled with a fluorescent protein.
      ***Note:*** In general, staining with an antibody provides better signal-to-noise ratio than using fluorescent protein labeling. Therefore, the BEDCA algorithm uses different parameters if one or both of the analyzed kinetochore markers is labeled with a fluorescent protein (see step 13 b. i.).
    viii. Save the changes to the Expt_Data_list file by simultaneously pressing **Command** and **S** on the keyboard.
13. Use of BEDCA troubleshooting 4
  Run BEDCA. In the command window type: Run_BEDCA(n, 'treatment', [], struct('filterdist', 200), struct( 'nsteps', value, 'a', 4)), where n is the experiment item number in the ExptData_list and ‘treatment’ is the name of the treatment to be analyzed. The number of steps the BEDCA algorithm will take in the run to infer the Delta distance is set by value following 'nsteps' (see 13 a V.). In the first instance a value of 100000 is suggested, i.e., 100,000 steps. The last two entries set options for BEDCA, so Run_BEDCA(n, 'treatment', [], [], []) runs BEDCA with default values. See the following for more information on the parameters and filters used.
  a. Parameters used by BEDCA
    i. Priors. The BEDCA algorithm uses priors (prior knowledge) for the spot spread in the x/y, and z directions, specifically for the precisions (inverse variances) taux (the noise is assumed to be the same in x and y) and tauz. Priors are needed as there is typically insufficient information in the data to use uninformed priors. Prior experimental knowledge, for instance, can be gleaned from Roscioli et al, 2020. The prior means for the standard deviations of the spot Gaussian in x (sdx, σx) and in z(sdz, σz) are set to 20 nm and 40 nm, respectively, for antibody-stained samples. As antibody staining in general results in better signal-to-noise ratio as compared to fluorescent protein labels, the sdx and sdz prior means are set to 25 nm and 75 nm, respectively, when one or both of the proteins are tagged with fluorescent protein. These prior means can be set by the user under the options structure, see help Run_BEDCA.
    ii. Alpha (a). The a parameter controls the prior variance; the relative error (of the prior) is 1/√a. A relative error of 30% (a=3) seems to work on the majority of the data, whilst 100% (a=1) is advised if possible. The a value by default is set to 4 and allows for 50% relative error in the taux and tauz prior. The user is recommended to use a low a value if possible, although to retain comparability across experiments using the same a is recommended.
    iii. The effect of your priors (means of taux, tauz, and a) should be checked post run. The parameter posterior distribution should lie well within the prior distribution, i.e., the data has improved on the estimates (from prior knowledge) whilst being consistent with that prior knowledge. See Figure 6.

14. BEDCA output troubleshooting 5
  a. Quality control: BEDCA Warning messages. BEDCA can alert the user of a problem by displaying in the Command window the following warnings:
    i. taux too strong or inappropriate and tauz too strong or inappropriate. The data isn’t consistent with your prior choices (either taux, tauz means or a). Check that the a parameter is set to 4 as default, and if a=4, try a smaller a, such as a=1. Check your prior estimates of spot center accuracy (mean taux, tauz). Alternatively, the user should check if the staining is annotated correctly, i.e., the input of ‘Ab’ or ‘GFP’ stain in the ExptData_list file. If neither of these resolves the warning, the user should go back to step 2 and optimize the imaging conditions such that the signal-to-noise ratio is improved.
    ii. Fewer than 300 KT. Algorithm performance poor especially for distances < 50nm and Effective number of cells is <5. Biological variation will not be averaged out, so estimates are unreliable. In that case, the user should perform another biological replicate, analyze it (KiDv1.0.1) and pool the data as described in step 9 above before running the BEDCA correction.
  b. Quality control: Evaluation of the BEDCA run.
    **CRITICAL:** The user should **always** evaluate the run to make sure the obtained results are informative.
    i. Evaluate the posterior Distribution. See Figure 7A. The distributions of the inflated (input) and corrected Delta (output) are automatically plotted and the figure is saved in MATLAB/MCMCrunsyAnalysisNameyFigures under the name ‘‘DataWithPostEsts’’. Here, AnalysisName refers to the name under which the analysis is saved (see step 12 b. v.). First, check that part of the distance distribution has not been cut off by the filtering of Delta 3D distances over 200nm (i.e., does not lie in the obvious tail of the distribution and is only removing what look like outliers). If the distribution has been cut off by the filter, change the filter as appropriate (see step 13 b. ii.) and rerun the algorithm again (see step 13). Second, check that the (uncorrected) distance distribution does not display more than one obvious peak. That would suggest a mixture of two distances which is inappropriate for BEDCA (we have not yet observed such case).

**Figure 8:**
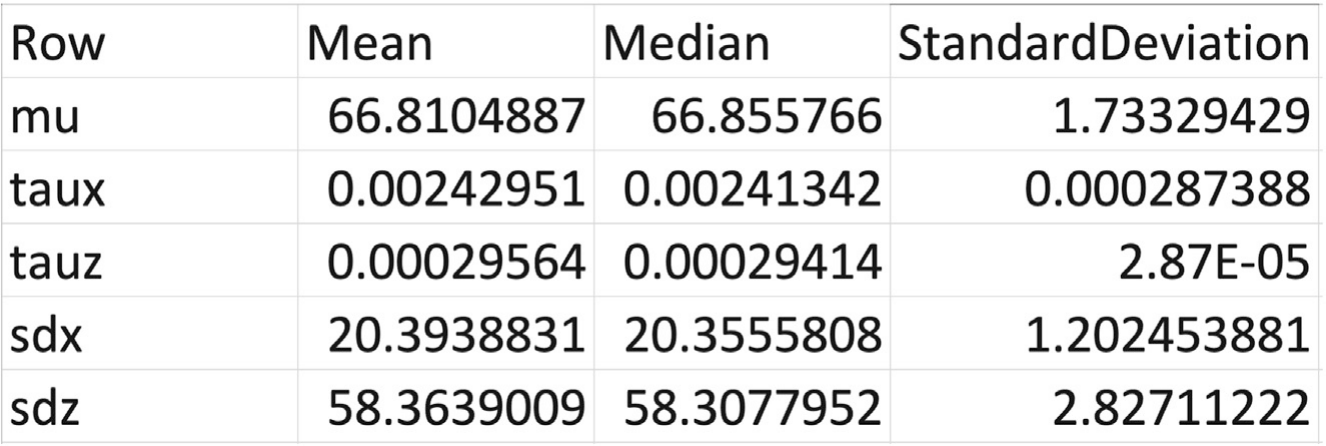
BEDCA output table illustrated with analysis of the Nnf1-to-Ndc80N distance from the exemplar dataset The table shown here illustrates the obtained parameters after analysis of the Nnf1-to-Ndc80N distance from the exemplar dataset, and the format of the output .csv file that the user can expect. In the .csv file, BEDCA automatically saves the mean, median and standard deviation of the following inferred parameters of the measured distance: the distribution mean (mu, nm), taux and tauz (nm^-2^, see step 14 b i.), sdx and sdz (nm). The .csv file has a general name of “SummaryStats_chain1” in MATLAB\MCMCruns\AnalysisName, where AnalysisName refers to the name under which the analysis is saved (see step 14 b. v.).
    iii. Kinetochore and cell sample size. The number of cells, number of kinetochores (KTs), average kinetochores per cell (average KTs/cell), number of kinetochore pairs (number pairs of KTs), and the effective cell sample size (Effective (cell) sample size) numbers of the analyzed dataset are stated at beginning of each BEDCA run in the Command Window. If the user wishes to display these characteristics again after the run, the user can type: preRunchecks(n, 'treatment', [], struct('filterdist', 200), struct ( 'nsteps', value, 'a', 4)), where the input of the function is the same as the one used in Run_BEDCA previously (see step 13). Here, BEDCA will only display the characteristics without running again the analysis.
      ***Note:*** Additional figures saved in the BEDCA run are depicted in Figures S3–S6.

### Making figures of cells and kinetochore pairs used in the analysis

**Timing: 30 min to 1 h for step 16**

**Timing: 15–30 min for step 17**

**CRITICAL:** For steps 16 and 17, the user is required to open again the KiDv1.0.1 folder in the Current folder panel (see step 3).

15. Plotting a kinetochore sister pair with at least one of the kinetochores used for analysis. In the command window type: dublShowSisterPair(mS_Name{i}, ‘sisterPair’, XX), where mS_Name is the structure created in step 6 or 9, i is the cell number, XX is the kinetochore pair number.
  a. Channels to be plotted. The user can specify which channels to be plotted by using option ‘channels’, [a], where a are the image channels to be plotted.
    **For example:**‘channels’, [1 2 3] will prompt KiDv1.0.1 to plot three of the imaged channels, while ‘channels’, [1 2] will prompt KiDv1.0.1 to plot the first and second acquired channel.
  b. Changing the image contrast.
    The input for the ‘contrast’ option is in the form of {[0.1 1] [0.1 1] [0.1 1]}, where each bracket corresponds to a fluorescent channel in the order 488 nm, 561 nm, 647 nm. The contrast input is in the borders between 0.1 and 1.
    Increase in the lower limit will increase contrast in the corresponding channel.
    **For example:**‘contrast’, {[0.1 1] [0.2 1] [0.1 1]} will increase the contrast in the 561 nm channel
    Decrease in the upper limit will lower the contrast in the corresponding channel.
    **For example:**‘contrast’, {[0.1 1] [0.1 1] [0.1 0.9]} will lower the contrast in the 647 nm channel.
  c. Plotting two images at the same intensity scale. The user can obtain the true intensity value of each fluorescent channel by using ‘contrast’, ‘help’. Next, the user needs to calculate by what percentage the contrast of the second image needs to be adjusted to match the first image intensity scale. **For example:** The first image intensity is 50 and the second image intensity is 100. To be on the same intensity scale, the intensity of the second image needs to be decreased by 50%. In the scale of 0.1 to 1 that corresponds to 0.5. Therefore, the user can plot the second image with option ‘contrast’, [0.1 0.5] for the respective channel and the two images will be on the same intensity scale.
    ***Note:*** after input of ‘contrast’, ‘help’, KiDv1.0.1 will prompt the user to choose contrast scale. If the user has not yet calculated the wanted scale, input of [0.1 1] will allow KiDv1.0.1 to continue to the next channel.
  d. Zoom. The user can choose to plot an image zoomed in at the kinetochore pair by using option ‘zoom’, 1 or to plot the full cell using option ‘zoom’, 0. In each of the cases KiDv1.0.1 will mark the detected kinetochore centers of the ‘sisterPair’.
  e. Z projection. By default, KiDv1.0.1 plots maximum intensity projection over five z slices surrounding the kinetochore pair center z-slice. The user can choose to plot a single Z slice containing the detected kinetochore pair center by using the option ‘zProject’, 0. Alternatively, the user can plot a maximum intensity projection of all z slices by using option ‘zProject’, 1, or a maximum projection of 5 slices total centered at the kinetochore-pair slice by using ‘zProject’, -1.
  f. Example. dublShowSisterPair(mS_exp1_13042020_Nnf1CenpCNdc80N_DMSO{1}, ‘sisterPair’, 5, ‘imageChans’, [1 2 3], ‘contrast’, {[0.1 1] [0.2 1] [0.1 1]}, ‘zoom’, 1, ‘zProject’, -1). With such stated options, KiDv1.0.1 will display the first movie of the Nnf1CenpCNdc80N analysis, the 5^th^ kinetochore pair as detected in the CS channel in all three fluorescent channels, the contrast is default for the 488 nm and 647 nm channels and enhanced in the 561 nm channel. Further, the image is a zoom-in at the kinetochore pair and represents a maximum intensity z-projection over 5 slices.

16. Plot one of the analyzed cells. In the command window type: kitShowImage(mS_Name{i}), where mS_Name is the structure created in step 6 or 9, and i is the cell number.
  ***Note:*** The additional options are inputted separated by commas as in step 15.
  a. Channels to be plotted. The user can specify which channels to be plotted by using option ‘imageChans’, [a], where a are the image channels to be plotted.
    **For example:**‘imageChans’, [1 2 3] will prompt KiDv1.0.1 to plot three of the imaged channels, while ‘imageChans’, [1 2] will prompt KiDv1.0.1 to plot the first and second acquired channel.
  b. Coordinate system. By default, KiDv1.0.1 will display the image in the x and y coordinates. The user can plot the x and z, or y and z coordinates by using option ‘coords’, ‘xz’ or ‘coords’, ‘yz’, respectively.
  c. Crop. By default, KiDv1.0.1 shows crop of the image corresponding to the ROI selected in the jobset set up. The user can display the full image or the full image with the selected crop annotated by using option ‘crop’, 0 or ‘crop’, -1, respectively.
  d. Projection range. By default, KiDv1.0.1 will not project over the third axis not given by ‘coords’. To make a maximum intensity projection, the user can use ‘projectionRange’, ‘help’. Next, KiDv1.0.1 will display the possible values the range can take in pixels and request a range from the user. The user should input the two values in square parentheses.
  e. Changing the image contrast.
    The input for the ‘contrast’ option is in the form of {[0.1 1] [0.1 1] [0.1 1]}, where each bracket corresponds to a fluorescent channel in the order 488 nm, 561 nm, 647 nm. The contrast input is in the borders between 0.1 and 1.
    Increase in the lower limit will increase contrast in the corresponding channel.
    **For example:**‘contrast’, {[0.1 1] [0.2 1] [0.1 1]} will increase the contrast in the 561 nm channel
    Decrease in the upper limit will lower the contrast in the corresponding channel.
    **For example:**‘contrast’, {[0.1 1] [0.1 1] [0.1 0.9]} will lower the contrast in the 647 nm channel.
  f. Plotting two images at the same intensity scale. The user can obtain the true intensity value of each fluorescent channel by using ‘contrast’, ‘help’. Next, the user needs to calculate by what percentage the contrast of the second image needs to be adjusted to match the first image intensity scale. **For example:** The first image intensity is 50 and the second image intensity is 100. To be on the same intensity scale, the intensity of the second image needs to be decreased by 50%. In the scale of 0.1 to 1 that corresponds to 0.5. Therefore, the user can plot the second image with option ‘contrast’, [0.1 0.5] for the respective channel and the two images will be on the same intensity scale.
    ***Note:*** after input of ‘contrast’, ‘help’, KiDv1.0.1 will prompt the user to choose contrast scale. If the user has not yet calculated the wanted scale, input of [0.1 1] will allow KiDv1.0.1 to continue to the next channel.
  g. Scale Bar. By default, KiDv1.0.1 will display scale bar of 3 μm at the bottom right of the image, without length label. To change the length of the scale bar use option ‘scaleBarSize’, x, where x is the desired length in μm. To display label of the scale bar length, use option ‘scaleBarLabel’, 1.
  h. Labeling on the image. To display text overlaid with the image use option ‘textNorthWest’, ‘textNorthEast’ or ‘textSouthEast’ to display the text in the upper left corner, upper right corner or bottom right corner, respectively. Input the text in the form {‘label’}, where label is the text, or to separate the text in multiple line use {‘label1’, ‘label2’, label3’} where each label will appear on separate line.
    **For example:**‘textNorthEast’, {‘Nnf1-A488’, ‘CenpC-A561’, ‘Ndc80(N)-A647’} will display in the upper right corner Nnf1-A488, CenpC-A561 and Ndc80(N)-A647, each on a new line.
  i. Transpose the image. To transpose the image, i.e., flip the image on its main diagonal axis, use option ‘transpose’, 1

17. Bibliography.
  a. Abbreviations in text:
    i. ACS – approximate chromatic shift
    ii. CS channel – coordinate system channel.
    iii. NCS – non-coordinate system channel.
  b. Variable functional name abbreviations
    i. jS – jobset, created in steps 3 and 4.
    ii. mS – movie structure, created in step 6 or 9.
    iii. sS_sel – spot selection **before** the metadata is updated to adhere to raw movie numbers, created in steps 6, 7, and 8
    iv. sS – spot selection **after** the metadata is updated to adhere to raw movie numbers, created in steps 6, 7, and 8
    v. iM – intra-measurements, file that contains intra-kinetochore Delta measurements, created in step 10.
    vi. IntiM – intensity intra-measurements, file that contains kinetochore intensity measurements, created in step 11. The kinetochore sample can be chosen to be the kinetochore subset used for intra-kinetochore Delta measurements, created in steps 6, 7 or 8.
  c. List of functions used and their syntax, refer to the protocol for complete information on the available options.
    i. jS_Name = kitGUI
    ii. dublManualPairSisters(jS_Name, ‘imageChans’, [x], ‘plotChan’, x, ‘coordChan’, vector)
    iii. sS_Name_sel = kitSelectData(mS_Name_sel, ‘channel’, x, ‘method’, ‘deselect’)
    iv. sS_Name= kitUpdateSpotSelelections(sS_Name_sel, mS_Name, SelectedCells_Name)
    v. sS_Name *=* kitFilterSpots(jS_Name, ‘channel’, x)
    vi. iM_Name = dublIntraMeasurements(mS_Name, ‘centralise’, 1, ‘channels’, [a b], ‘spotSelection’, sS_Name)
    vii. dublBasicStats(iM_Name)
    viii. dublBasicPlots(iM_Name)
    ix. IntiM_Name = dublIntensityMeasurements(mS_Name, ‘channels’, [a b], ‘paired’, 0 or 1, ‘refMarker’, ‘option’, ‘spotSelection’, sS_Name)
    x. sS_Pool_Name = kitCombineSpotSelections({sS_Name, sS_Name, sS_Name})
    xi. Run_BEDCA(n, 'treatment', [], struct(‘filterdist’, 200), struct( 'nsteps', value))
    xii. preRunchecks(n, 'treatment', [], struct(‘filterdist’, 200), struct( 'nsteps', value))
    xiii. dublShowSisterPair(mS_Name{i}, ‘sisterPair’, XX)
    xiv. kitShowImage(mS_Name{i})

## Expected outcomes

After successful running of the KiDv1.0.1. and BEDCA software with the exemplar dataset, the user is expected to obtain mean Euclidian corrected distance between Nnf1 and Ndc80N close to 66 nm, with standard deviation close to 2 nm. The results that we obtain after analysis of the exemplar dataset Nnf1-to-Ndc80N distance are shown in Figure 8. This result is automatically saved after the run in a .csv file. The user can access the .csv file of their analysis as described in step 14 b i. and compare it to the one displayed in Figure 8. If the results are more than 2 nm different, the user is suggested to revise the spot selection criteria, and to check if the BEDCA run has passed all of the checks stated in step 14 a.

## Limitations

The user should not use the above protocol for analysis of structures that produce non-Gaussian shaped staining. For example, kinetochore components that have formed crescents. The KiDv1.0.1 software cannot detect the center of crescents accurately, and thus the results are unreliable.

## Troubleshooting

### Problem 1

Metadata not read in correctly or at all (steps 3 and 4).

### Potential solution

Open the image via BioFormats import in Fiji, do not use virtual hyperstack here. Once opened, the image should have the correct Z sectioning and channels. If not, re-order the movie and save it as ome.tif. If the problem persists, make sure you followed steps 1 and 2 accurately.

### Problem 2

No or very few spots detected in the majority of jobs (image stacks) (steps 3 and 4).

### Potential solution

In our imaging set up, KiDv1.0.1 signal-to-noise ratio higher than 2. However, that range may be slightly different depending on the imaging set up. To deduce the range for your system, image metaphase cells with different exposure and laser power. Use appropriate ACS as described in steps 1–4. Analyze the cells as suggested in step 4 and note which conditions give close to the expected number of spots (KiDv1.0.1 will display in the Command window number of spots detected in each movie). Use the condition with best quality spots from the ones with acceptable amount of detected spots.

**CRITICAL:** Always inspect the spots visually. If the shape of the spot is not clearly distinguished from the background or if the spot shape is not Gaussian (see images throughout the protocol and example dataset for examples of the quality of spots expected), the signal of the sample is insufficient or the marker is not appropriate for this protocol.

***Note:*** If more than one channel satisfies the criteria of coordinate system channel, the user can also try to switch the channel used for coordinate system channel and check if the detection improves. This may help in cases where, albeit non-apparent, the background is not registered properly when using a specific antibody.

**CRITICAL:** If none of the above suggestions works, the user is highly recommended to perform the experimental control from the “Before you begin section” to exclude technical issues.

### Problem 3

“createDistanceMatrix.mexmaci64” cannot be opened because the developer cannot be verified (step 5).

### Potential solution

Open the “System Preferences” on the computer. In the “Security and Privacy” section, the user will see a notice of: “createDistanceMatrix.mexmaci64 was blocked from use because it is not from an identified developer”. Next to the message, an Allow Anyway button will be displayed. Click on the button with the left mouse button.

***Note:*** If the button cannot be clicked, make sure that the lock at the bottom is open.

### Problem 4

'Run_BEDCA′ is not found in the current folder or on the MATLAB path, but exists in: …” (step 14)

### Potential solution

To run BEDCA, the Current Folder should be set to the BEDCA folder, containing the code. If the contents of the “BEDCA” folder do not display in the Current Folder panel of MATLAB, navigate to the BEDCA folder in the “Current Folder” panel.

### Problem 5

Convergence of a pooled dataset is not achieved despite large kinetochore number and good quality data (step 14).

### Potential solution

Check the convergence and BEDCA parameters of the individual experiments. If one stands out, check for technical problems with that particular experiment. Convergence can only be achieved if the input distributions are similar, i.e., all are sampled from the same distribution. If due to technical reasons, one of the experiments is an outlier, convergence will not be achieved. In our experience, this was typically due to failure of treatment. If this is not the case, we recommend that the user checks for other sources of biological variability.

## Resource availability

### Lead contact

Further information and requests for resources and reagents should be directed to and will be fulfilled by the lead contact, Nigel Burroughs (N.J.Burroughs@warwick.ac.uk).

### Materials availability

This study did not generate new unique reagents.

## Data Availability

This study did not generate new code and did not analyze new datasets.
