# Supplementary material for: Subcellular Euclidean distance measurements with multicolor fluorescence localization imaging in cultured cells
Source: STAR Protoc. 2021 Nov 15;2(4):100774. doi: 10.1016/j.xpro.2021.100774 (PMC8605398; doi:10.1016/j.xpro.2021.100774)
Supplement: Document S1. Figures S1–S6 [file mmc1.pdf]

A.

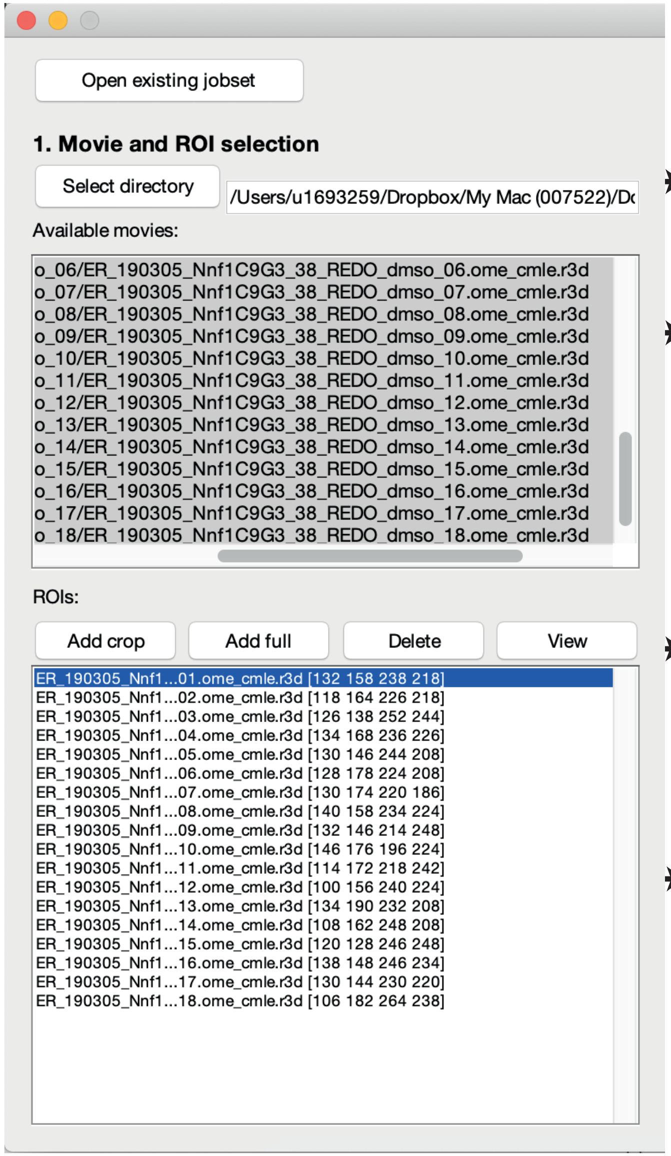

Click on Select directory and choose the folder containing the raw data

The Available movies (image stacks) will appear here. Left-click with the mouse on a movie to select it.

Click Add full to enter the full image (for ACS analysis) or Add crop to analyse only the region containing the cell of interest (the ROI, for Sample analysis), see bellow.

The user can select a ROI and click Delete to delete it and View to view it.

If Add crop is clicked, Window with maximum Z-projection of the selected image stack will appear.

Use the left mouse button to make a rectangle around the cell of interest. Double-click with left mouse button on the rectangle to confirm it (it will become yellow), see example on the right.

Click Finish.

The added ROI will now appear in the ROIs window

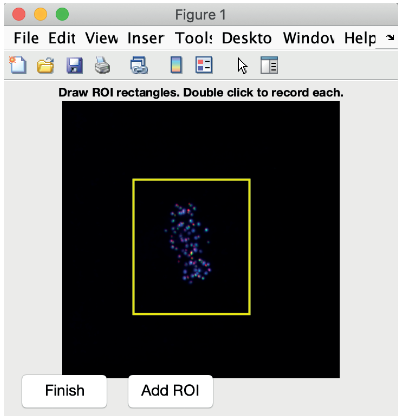

B.

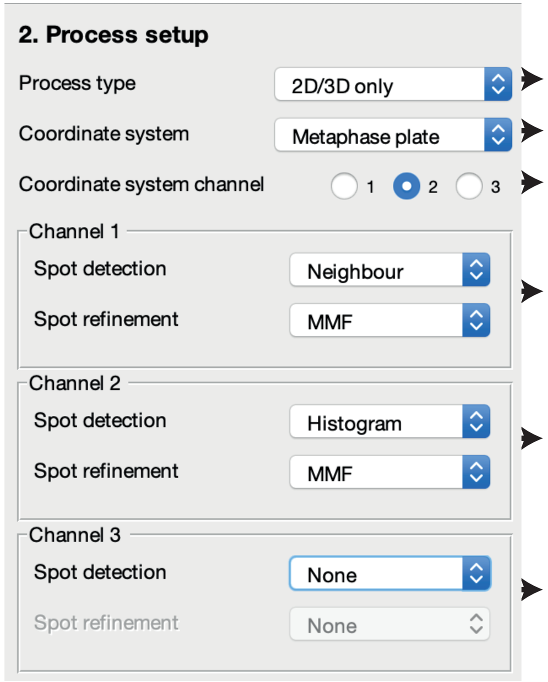

Select 2D/3D only. for sample analysis and Chromatic shift for ACS analysis

Choose Metaphase plate if the kinetochores are aligned and Centre of mass otherwise.

Select coordinate system channel based on criteria in step 7 c iii. Numbers relate to order of aquisition.

Set the detection to Neighbour and the refinement to MMF if the channel is non-coordinate system channel and contains kinetochore marker staining.

Set the detection to Histogram and the refinement to MMF if the channel is coordinate system channel.

Set the detection to None if the there is no kinetochore marker imaged.

C.

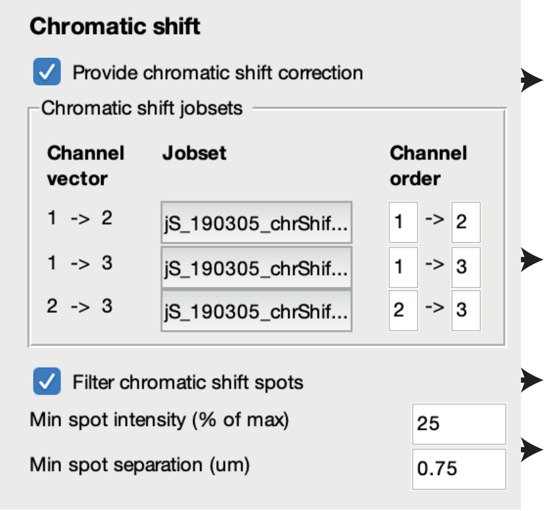

Click on the box to Provide chromatic shift correction for Sample analysis.

Click on the one of the boxes bellow Jobset to provite the ACS jobset created in step 4. Load the created jobset for all used channel combinations (channel vectors).

Click on the box to Filter chromatic shift spots

Set the Min spot intensity to 25 , i.e. 25% from the max intensity in the image. Set the Min spot separation to 0.75 µm - the resting inter-sister distance.

Click on the blue arrows to see the available options

Supplemental Figure 1. Kinetochore Delta (KiDv1.0.1) software graphic user interphase (GUI), Related to step 13. Instructions on: A) Entering imaging data in KiDv1.0.1. B) Spot detection process set up for the acquired imaging channels. C) Set up of chromatic shift correction. For full details see steps 13 and 14 from the protocol.

A.

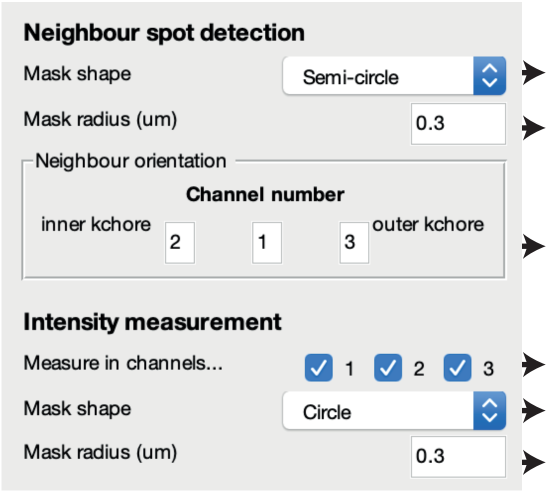

Indicates the mask shape in which KiD will scan for spots starting with the ACS-transformed spot coordinates of the CS channel and searching in the channels where Neighbor spot detection is specified. Semi-circle uses the detected plate to inform direction of the mask in semi-circle outwards of the plate. Circle denotes circle mask around the spot in the CS channel coordinates.

The mask radius indicates in what radius KiD will scan for spots in the neighbour NCS channel starting from the ACS transformed spot coordinates in the CS channel. The default is 0.3 micrometer, chosen as kinetochores are largerly diffraction-limited spot, i.e. with size bellow 250nm.

Indicate order of channels based on approximate location of the kinetochore markers relative to each other. Results are not affected by the order. Note the chosen order.

Click on the boxes next to the channels for which Intensity measurement is desired.

Intensity is measured in the indicated mask shape around the coordinates of the spot in CS channel transformed after ACS in the neighbour NCS channel. The default option is Circle but Semi-circle and Cone are also available as options.

The mask radius indicates in what radius KiD will measure the spot intensity in the neighbour NCS channel starting from the ACS transformed spot coordinates in the CS channel. The default is 0.3 micrometer, chosen for the reason stated above.

B.

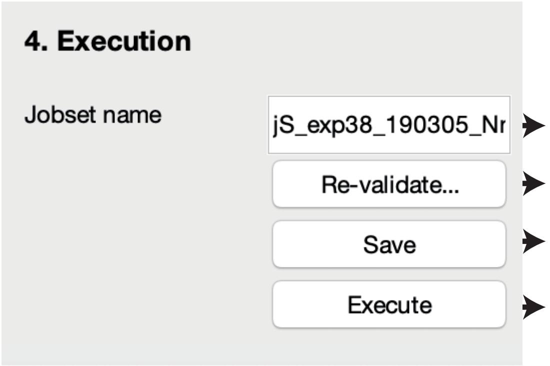

Type the Jobset name as chosen in 3a or 4a.

Click on Validate. A window with the image stack metadata will pop-up. If the metadata is correct, click on the box next to Apply to all movies and then click Validate. Otherwise make sure the data is exported as stated in step 1 and 2.

Press Save to save the Jobset and run the analysis at a later time.

Press Execute to run the analysis. The Jobset is now automatically saved in the folder with raw data.

Supplemental Figure 2. Kinetochore Delta (KiDv1.0.1) software graphic user interphase (GUI), Related to step 13. Instructions on: A) Set up of parameters for Neighbour spot detection and intensity measurements. B) Saving and executing the jobset analysis.

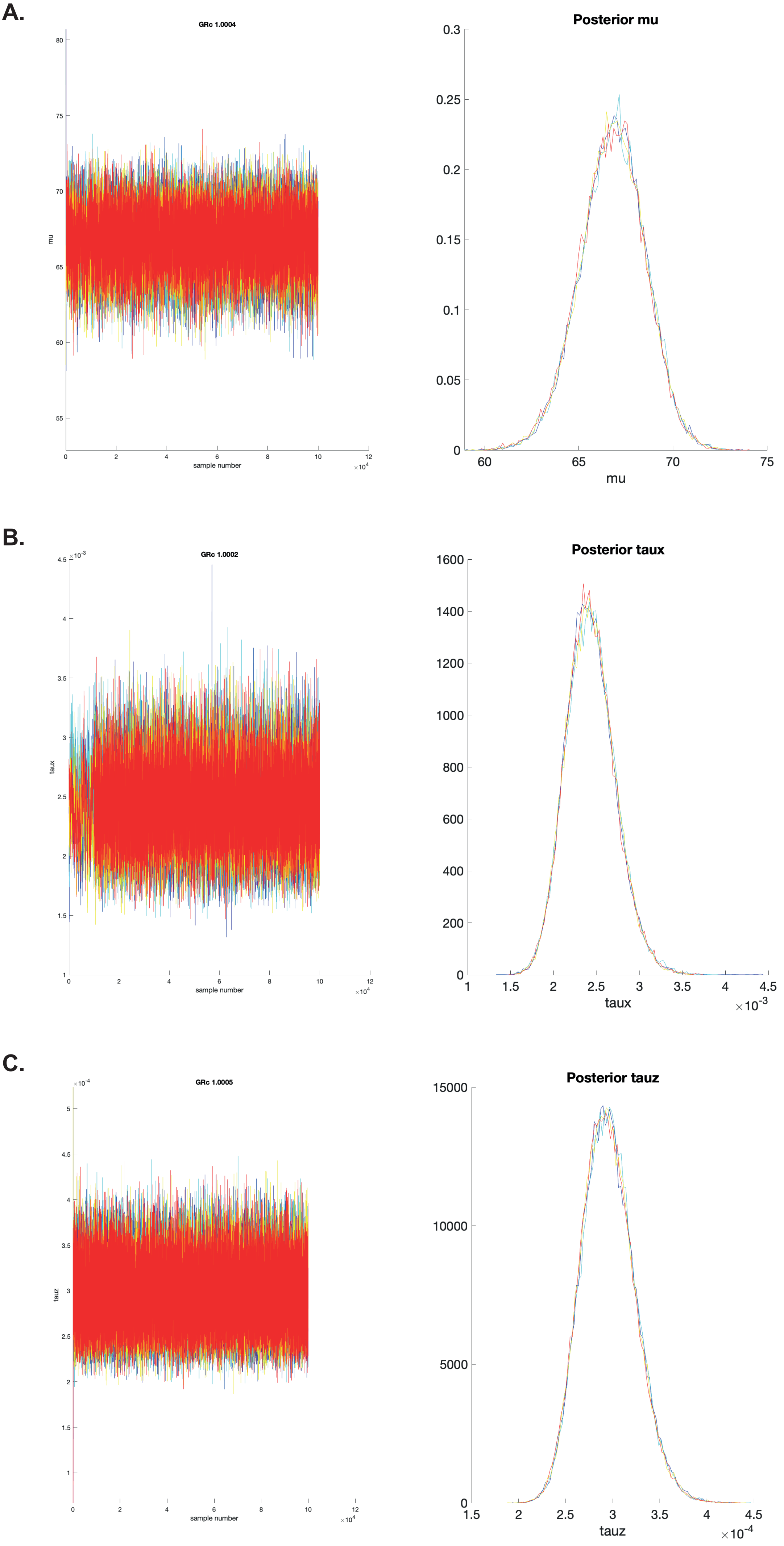

Supplemental Figure 3. Posterior distributions and samples of mu (nm), taux (nm^{-2}) and tauz (nm^{-2}), Related to step 25. A) Example of figure saved as “Posterior_mu” in “MCMC_EuclDistMargFigs-Conv” folder, using Nnf1-to-Ndc80N distance correction from provided dataset. Left: mu estimation at each sample, GRc shown above is below 1.01 and indicates convergence. Colors indicate the four runs. Right: Histogram of corrected mean (mu) in each of the four runs (red, yellow, dark and light blue). B) Example of figure saved as “Posterior_taux” in “MCMC_EuclDistMargFigs-Conv” folder, dataset as in A. Left: taux (precision in x) estimation at each sample, GRc shown above is below 1.01 and indicates convergence. Red, yellow, dark and light blue colours indicate the four runs. Right: Histogram of corrected taux (precision in x) in each of the four runs (red, yellow, dark and light blue). C) Example of figure saved as “Posterior_tauz” in “MCMC_EuclDistMargFigs-Conv” folder, dataset as in A. Left: taux (precision in z) estimation at each sample, GRc shown above is below 1.01 and indicates convergence. Red, yellow, dark and light blue colours indicate the four runs. Right: Histogram of corrected taux (precision in z) in each of the four runs (red, yellow, dark and light blue).

A.

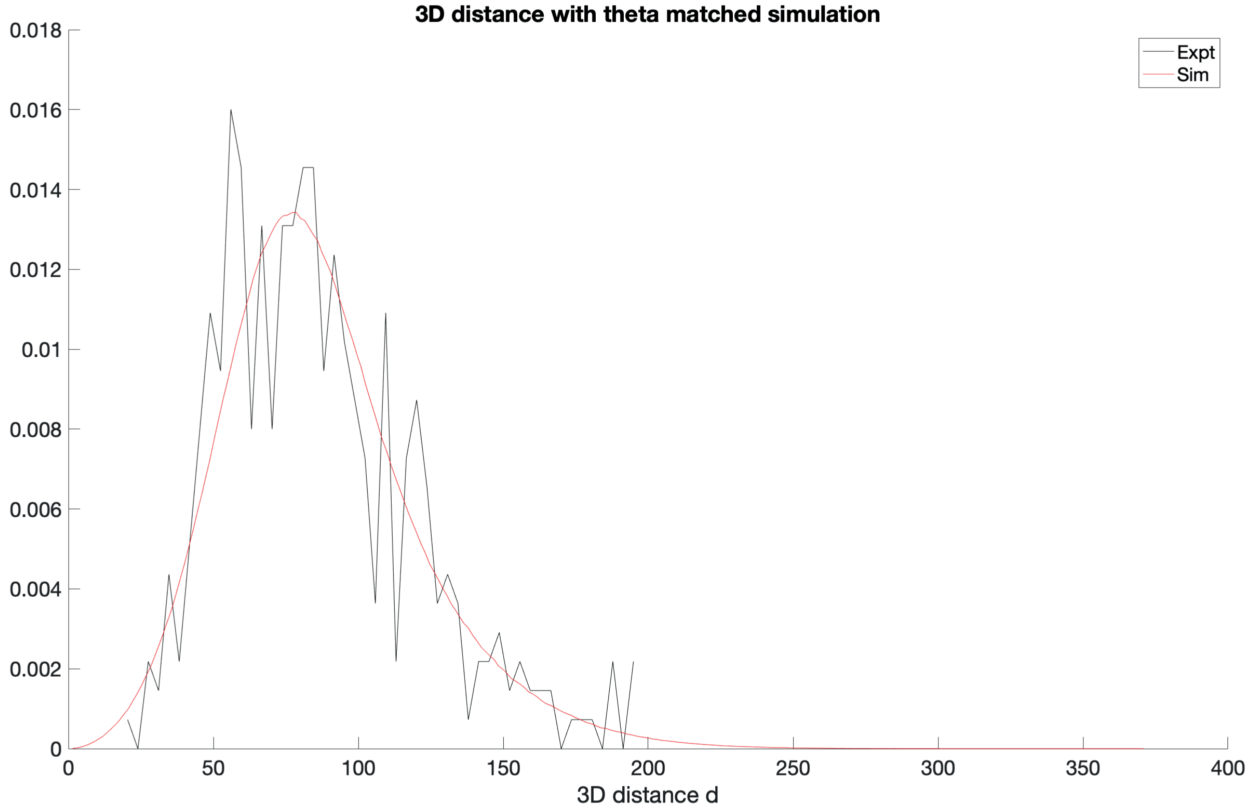

B.

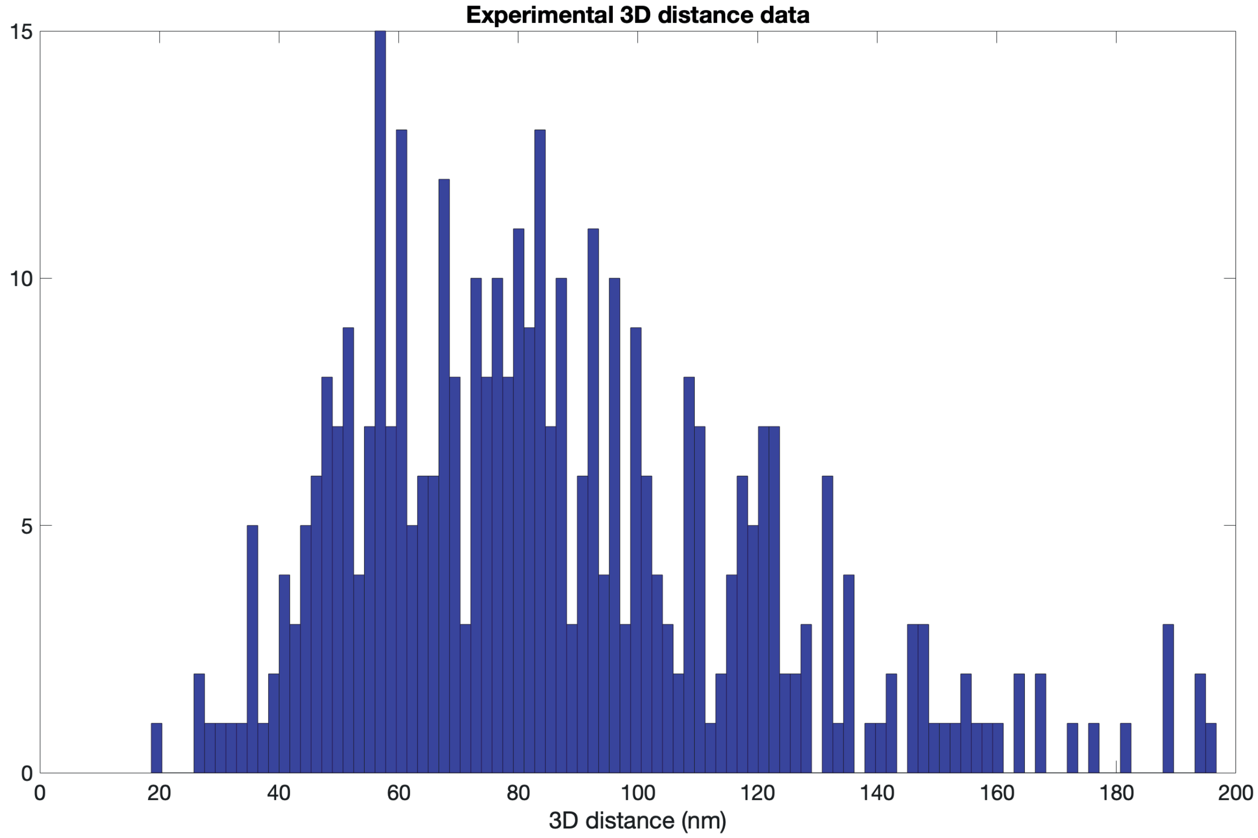

Supplemental Figure 4. ∆3D distance distributions and simulation, Related to step 25. A) Example of figure saved as "DataSimComp" in "Figures" folder, using Nnf1-to-Ndc80N distance correction from provided dataset. Histogram of the measured 3D delta distance distribution (black, nm) and the simulated 3D delta distribution in BEDCA after sampling. B) Example of figure saved as "MCMC_EuclDistMarg3DrData", dataset as in A. Histogram of the 3D delta distance measurements that are input in BEDCA.

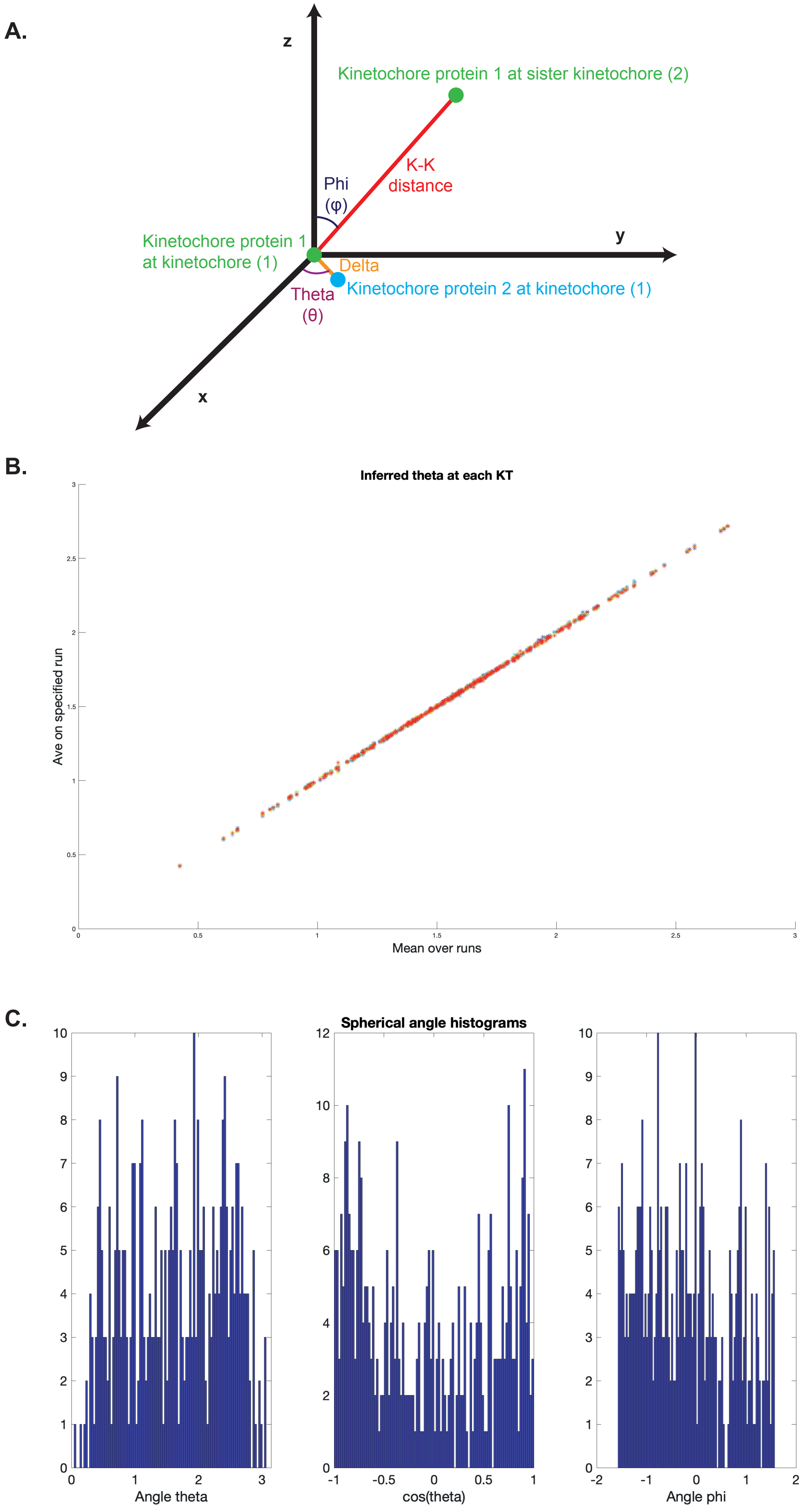

Supplemental Figure 5. Kinetochore-Kinetochore distance and Intra-kinetochore Delta distance spherical angles, Related to step 25. A) Schematic showing the theta and phi angle annotation as related to K-K and Delta vector angles inferred by BEDCA. B) Example of figure saved as "ConvPosterior_hiddenstates" in "MCMC_EuclDistMargFigsConv" folder, as automatically saved for Nnf1-Ndc80N measurement from the provided dataset. The figure shows average inferred theta per run (red, yellow light and dark blue) versus the mean of inferred theta over the four runs. C) Example of figure saved as "MCMC_EuclDistMarg_angularData", dataset as in B. The figure shows histograms of the inferred theta, cosine(theta) and phi (see A.).

A.

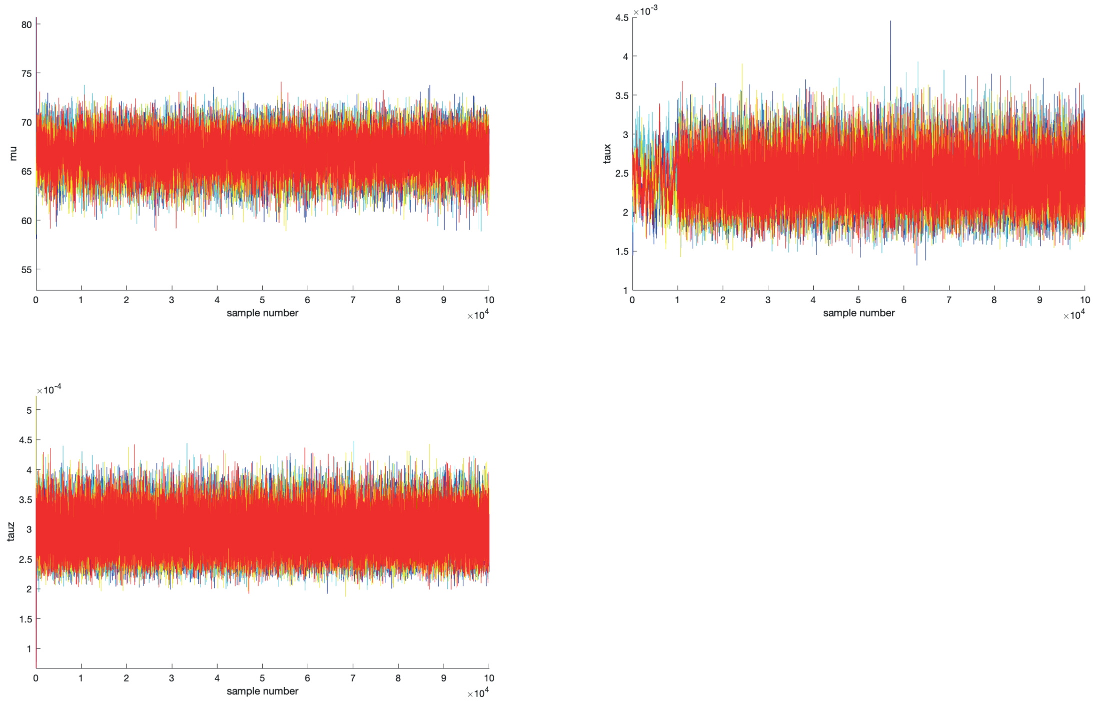

B.

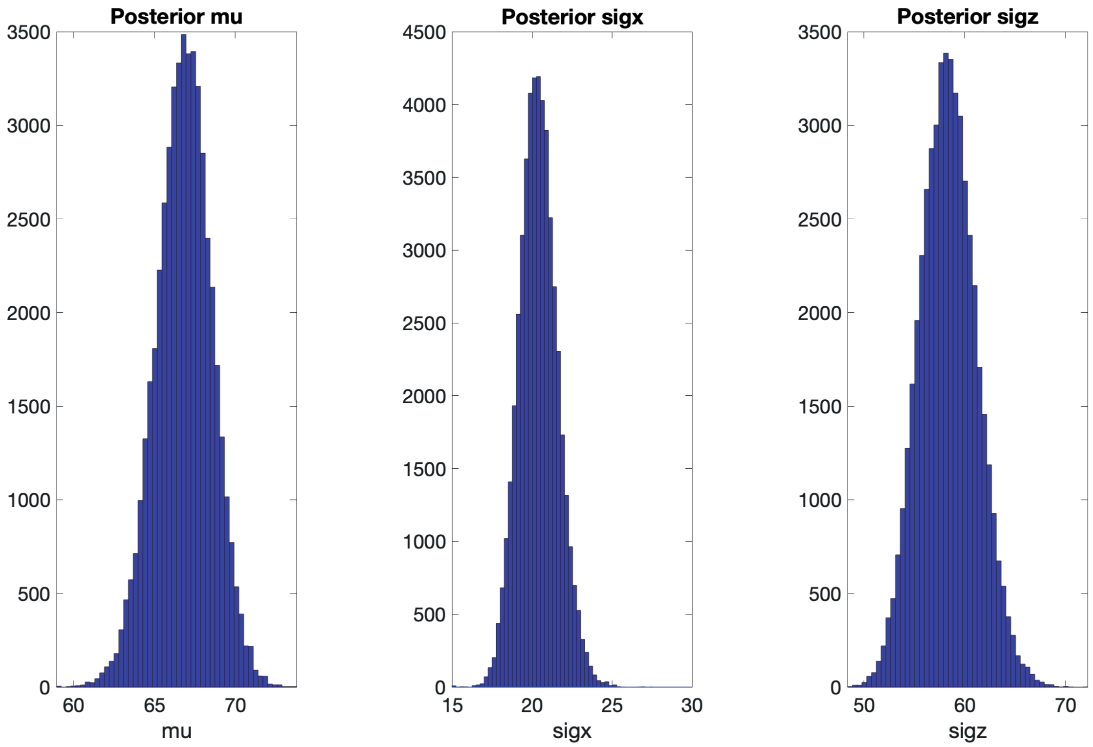

Supplemental Figure 6. ΔEC mean (nm), taux (nm^{-2}) and tauz (nm^{-2}) inference per sample per run and histograms of the posteriors, Related to step 25. A) Example of figure saved as “DataSimComp” in “Figures” folder, using Nnf1-to-Ndc80N distance correction from provided dataset. Inference of the mean (mu), taux (precision in x) and tauz (precision in z) at each sample for each run (red, yellow, light and dark blue). B) Example of figure saved as “PosteriorParams” in “Figures” folder, dataset as in A. Histogram of estimated posterior distribution of mu (nm), sigx (nm, standard deviation of spot gaussian in x), and sigz (nm, standart deviation of spot gaussion in z), respectively left to right. X axis values are shown in nm.
